# Amygdala neuronal dyshomeostasis via 5‐HT receptors mediates mood and cognitive defects in Alzheimer's disease

**DOI:** 10.1111/acel.14187

**Published:** 2024-05-08

**Authors:** Xin‐Rong Wu, Xiao‐Na Zhu, Yuan‐Bo Pan, Xue Gu, Xian‐Dong Liu, Si Chen, Yu Zhang, Tian‐Le Xu, Nan‐Jie Xu, Suya Sun

**Affiliations:** ^1^ Department of Neurology Institute of Neurology, Ruijin Hospital, Shanghai Jiao Tong University School of Medicine Shanghai China; ^2^ Department of Anatomy and Physiology, Collaborative Innovation Center for Brain Science Shanghai Jiao Tong University School of Medicine Shanghai China; ^3^ Department of Neurosurgery, Shanghai Ninth People's Hospital Shanghai Jiao Tong University School of Medicine Shanghai China; ^4^ Songjiang Hospital and Songjiang Research Institute Shanghai Jiao Tong University School of Medicine Shanghai China; ^5^ Key Laboratory of Cell Differentiation and Apoptosis of the Chinese Ministry of Education Shanghai Jiao Tong University School of Medicine Shanghai China; ^6^ Shanghai Key Laboratory of Emotions and Affective Disorders Shanghai Jiao Tong University School of Medicine Shanghai China

**Keywords:** 5‐HT receptors, Alzheimer's disease, basolateral amygdala, mood and cognitive defects, neuronal hyperactivity

## Abstract

Behavioral changes or neuropsychiatric symptoms (NPSs) are common features in dementia and are associated with accelerated cognitive impairment and earlier deaths. However, how NPSs are intertwined with cognitive decline remains elusive. In this study, we identify that the basolateral amygdala (BLA) is a key brain region that is associated with mood disorders and memory decline in the AD course. During the process from pre‐ to post‐onset in AD, the dysfunction of parvalbumin (PV) interneurons and pyramidal neurons in the amygdala leads to hyperactivity of pyramidal neurons in the basal state and insensitivity to external stimuli. We further demonstrate that serotonin (5‐HT) receptors in distinct neurons synergistically regulate the BLA microcircuit of AD rather than 5‐HT levels, in which both restrained inhibitory inputs by excessive 5‐HT_1A_R signaling in PV interneurons and depolarized pyramidal neurons via upregulated 5‐HT_2A_R contribute to aberrant neuronal hyperactivity. Downregulation of these two 5‐HT receptors simultaneously enables neurons to resist β‐amyloid peptides (Aβ) neurotoxicity and ameliorates the mood and cognitive defects. Therefore, our study reveals a crucial role of 5‐HT receptors for regulating neuronal homeostasis in AD pathogenesis, and this would provide early intervention and potential targets for AD cognitive decline.

## Graphical abstract

Mechanism scheme for exacerbating mood and cognitive deficits in Alzheimer's disease by dysregulation of 5‐HT receptors in BLA neurons. Aberrant dual serotonin receptors signaling integrates BLA neuronal function and mood defects in AD animal model: both increasing of pyramidal neurons its depolarizing function by upregulated 5‐HT_2A_R and indirect weakening of PV interneurons inputs via excessive 5‐HT_1A_R. Long‐term downregulation of these two serotonin receptors simultaneously ameliorates the mood and cognitive defects of AD and alleviates the Aβ neurotoxicity for neurons. Please see text for more details.

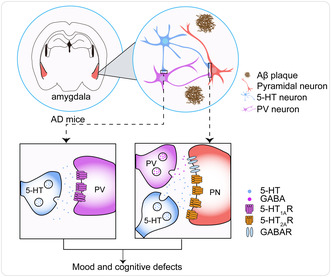

**Abbreviations**
5‐HT: serotonin
AD: Alzheimer's disease
BLA: basolateral amygdala
MCI: Mild cognitive impairment
NPSs: neuropsychiatric symptoms
PV: parvalbumin

## 1 INTRODUCTION

Alzheimer's disease (AD) is the leading cause of dementia, which is characterized by the gradual deterioration of memory. Currently, there are limited treatment options available once patients are diagnosed (Glazner & Kaplan, 2018). Mild cognitive impairment (MCI) serves as an intermediate stage between normal cognition and dementia, and individuals with MCI have a higher rate of progression to AD in a relatively short period of time (Albert et al., 2011). Behavioral changes or neuropsychiatric symptoms (NPSs) are common in MCI or AD patients, and are associated with more rapid functional decline to severe dementia (Ismail et al., 2016; Peters et al., 2015). Clinical and preclinical studies support the psychological perspective that mood and cognition are closely intertwined. Cognitive processing requires emotional responses, which is elicited to modulate perception and sensation in response to the environment (Brosch et al., 2013). Furthermore, substantial evidence supports that an abnormal mood state significantly increases the presence of molecular markers associated with the progression of AD (Sotiropoulos et al., 2008), while rescuing the initial mood disorders by antidepressants preserves the eventual memory function in AD mice (Ai et al., 2020). Previous neuroimaging studies have found that the NPSs are associated with specific regions of the brain, and these regions are connected and interact with each other, ultimately presenting as pathological symptoms (Chen et al., 2021). Although these pieces of evidence anatomically reveal possible mechanisms of mood and cognition interactions in brain regions, the association between NPSs and memory loss are linked in AD progression at the cellular and molecular levels remains poorly understood.

Among numerous hypotheses that have been proposed regarding the pathogenesis of AD, the abnormal hyperactivity of neurons and disruption of large‐scale brain networks are suggested as early signs of neuronal dysfunction in AD, which give rise to later cognitive impairments (Busche et al., 2019; Busche & Konnerth, 2015; Zott et al., 2019). Recent research reveals that abnormality in the 5‐HTergic pathway from the medial raphe nucleus to the hippocampus lead to hyperexcitability of CA1 pyramidal neurons and cognitive decline in AD mice (Wang et al., 2023). This is consistent with our previous research that neuronal calcium dyshomeostasis causes hyperactivity and could further induce dendritic spine loss (Sun et al., 2014; Zou et al., 2022). However, these studies are restricted to memory‐related nuclei such as the hippocampus and cortex, while lose sight of whether NPSs are also associated with excitability imbalance of neurons in these nuclei. Amygdala, an emotional brain region, is critically involved in the processing of emotional information that has widespread reciprocal connections with many cognitive regions, such as memory‐related regions (hippocampus) and perceptual pathways (primary visual cortex and inferior temporal cortex) (Choi et al., 2021; Tye et al., 2011). We thus hypothesize that aberrant neuronal activity occurs in emotional brain region such as amygdala, and the abnormality contributes to NPSs and memory decline during AD.

In the present study, we find that young adult APP/PS1 mice exhibit mood disorders before the onset of cognitive decline, and the basolateral amygdala (BLA) is involved in the pathological process. We observe that BLA neurons are hyperactive in their basal state and insensitive to external stimuli, leading to NPSs and memory loss as the AD progresses. Furthermore, we identify 5‐HT_2A_R in pyramidal neurons and 5‐HT_1A_R in PV interneurons within the BLA that synergistically maintain the homeostasis of neural microcircuits. Down‐regulating the expression of two 5‐HT receptors in the BLA rescues mood and memory deficits in AD model mice. In summary, our findings highlight the synergistic role of 5‐HT receptor subtypes in mediating BLA neuronal function during the pathogenesis of AD, suggesting their potential as early intervention targets for NPSs and cognitive impairment.

## 2 RESULTS

### 2.1 BLA neurons respond aberrantly in early‐stage AD

**FIGURE 1:**
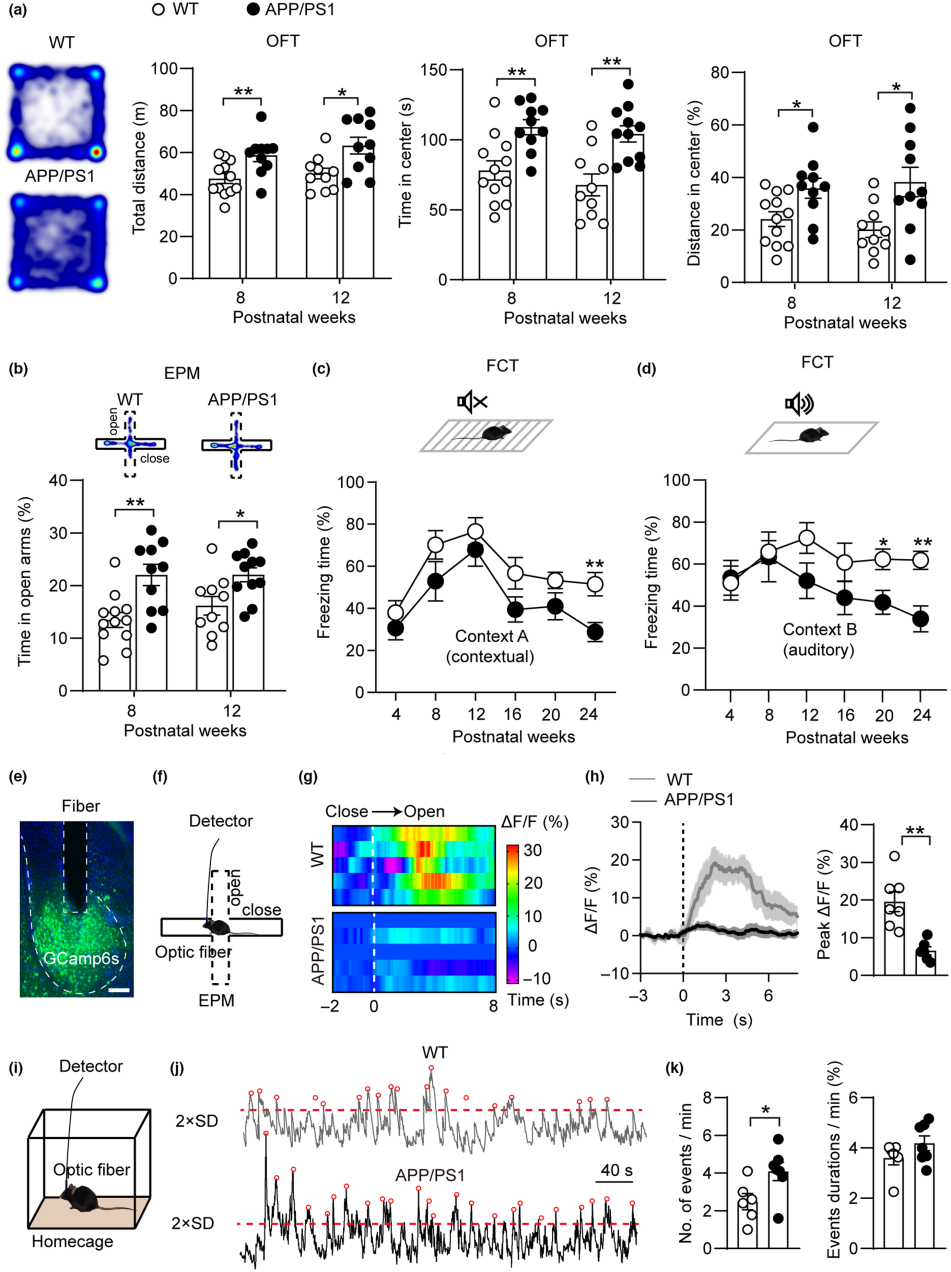
BLA neurons respond aberrantly in in early‐stage AD. (a) APP/PS1 mice showed longer time in center area and more total distance in open field test (OFT) compared to WT mice at PW8 and PW12 (*n* = 8–12 mice for each group). (b) APP/PS1 mice displayed an increased time in open arms compared to WT mice in elevated plus maze (EPM) at PW8 and PW12 (*n* = 8–12 mice for each group). (c, d) Time courses for freezing time of WT and APP/PS1 mice in context A and context B of fear conditioning test (FCT) (*n* = 8–13 mice for each group). (e) Representative image for viral expression and location of the optical fiber tract in the BLA. Scale bar: 200 μm. (f) Paradigm of the fiber photometry setup in the EPM. (g) Heatmaps illustrating the calcium response (ΔF/F) of BLA neurons in WT mice (upper panel) and APP/PS1 mice (bottom panel) when mice moved from closed arms to open arms in EPM. (h) Plot of averaged calcium signals during the EPM test onto open arms in WT (black) and APP/PS1 mice (gray). The solid line and the shaded regions are the mean ± SEM. The right panel showed the quantification of the peaks (ΔF/F) of calcium signals (*n* = 6–7 animals for each group). (i) Paradigm of the fiber photometry setup in the home cage condition. (j) Representative traces of population calcium activity in BLA neurons. (k) Number of events (>2 SD) in 1 min (left panel) and the percentage of event durations in 1 min (right panel) of calcium activity (*n* = 6–7 animals for each group). Significance was assessed by unpaired Student's *t* test in (a)–(d), (h) and (k). All data are presented as mean ± SEM. *p < 0.05; **p < 0.01.

To clarify the onset time of NPSs and cognitive impairment in the course of AD, we first assessed emotional and cognitive behaviors by using an AD mouse model and found that the young APP/PS1 mice showed abnormal behaviors, including longer total distance, increased center exploration in the open‐field test (OFT), and more time in the open arms in the elevated plus maze (EPM) paradigms at postnatal weeks 8 (PW8) and PW12, while the 24‐week‐old APP/PS1 mice showed memory impairment in the fear conditioning test (FCT) (Figure 1a–d). These data support the clinical observations that neuropsychiatric symptoms emerge much earlier than cognitive impairment during AD pathological processes.

To identify the potential brain regions both involve in aberrant emotional behavior and later cognitive defects in AD progression. We used TRAP (targeted recombination in active populations) technology to label the neurons in the EPM test at PW10 and stained c‐Fos after auditory FCT at PW24 (Figure S1a,b). The TRAP system permits the tagging of neurons transiently activated during a specific time window by using the Ai9‐Tdtomato reporter line crossed with *Fos* ^iCreERT2^ mice (Guenthner et al., 2013), which allows us to label neurons responsive to both emotion‐associated behaviors in the early stage of AD and cognitive function later on. We found that neuronal activity of the BLA is significantly downregulated both in emotional and cognitive behaviors, and fewer “emotional” neurons of the BLA respond to cognitive process during the AD course (Figure S1c–e). Due to the fact that lesions in the amygdala occur the prodromal stages of AD (Markesbery et al., 2006; Poulin et al., 2011; Wachinger et al., 2016) and its important role in regulating mood and memory under physiological and pathological conditions (Ehrlich et al., 2009; Phelps & LeDoux, 2005), we speculate that the amygdala is involved in regulating abnormal emotional and cognitive behaviors in AD.

To understand the BLA neuronal activity underlying aberrant emotional behavior changes, we used fiber photometry by implanting an optic fiber into the BLA in which AAV‐hSyn‐GCaMP6s was injected, which allowed us to record Ca^2+^ signals of neurons in freely moving mice (Figure 1e). We observed the dynamic changes in BLA neuronal activity during the EPM trial of PW10 mice and found a robust peak of Ca^2+^ signals when the WT mice explored from the closed arm to the open arms, while the signals of APP/PS1 mice remained unchanged (Figure 1f–h). Under home cage conditions, the Ca^2+^ activity of BLA neurons in AD mice was significantly higher than that in WT mice (Figure 1i–k). Consistent with Ca^2+^ activity, more c‐Fos was detected in the BLA of naïve APP/PS1 mice. However, the number of c‐Fos^+^ cells in the BLA of APP/PS1 mice was much lower than that in WT mice after exposure to the EPM (Figure S2). These data suggested that BLA neurons exhibit hyperactivity in the basal state and are blunt to external stimuli in early‐stage AD.

### 2.2 The imbalance of neuronal excitation and inhibition occurs in the BLA of early‐stage AD

**FIGURE 2:**
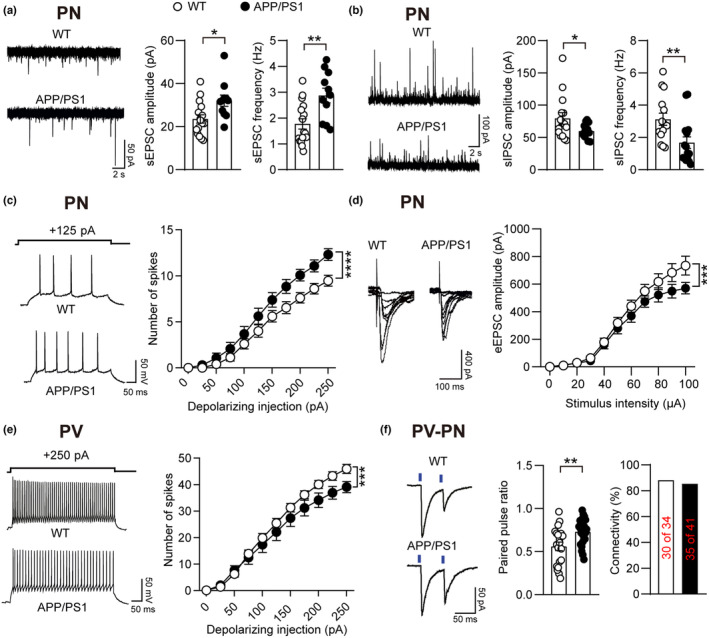
The imbalance of neuronal excitation and inhibition occurs in the BLA of early‐stage AD. (a) Sample traces of sEPSCs and statistical data for the amplitude and frequency of sEPSCs recorded from pyramidal neurons in the WT and APP/PS1 groups (*n* = 15 cells from 3 mice for each group). (b) Sample traces of sIPSCs and statistical data for the amplitude and frequency of sIPSCs recorded from pyramidal neurons in the WT and APP/PS1 groups (*n* = 15 cells from 3 mice for each group). (c) Representative traces of action potential firing and statistical data from pyramidal neurons in the WT and APP/PS1 groups (*n* = 13–15 cells from 3 to 4 mice for each group). (d) Representative traces of eEPSCs and statistical data from pyramidal neurons in the WT and APP/PS1 groups (*n* = 16–17 cells from 3 to 4 mice for each group). (e) Representative traces of action potential firing and statistical data from PV interneurons in the WT and APP/PS1 groups (*n* = 13–15 cells from 4 mice for each group). (f) Representative traces and quantification of PPR from the PV to PN in WT and APP/PS1 mice (*n* = 20–24 cells from 3 to 5 mice for each group). Statistical significance was assessed by two‐tailed unpaired Student's *t* test in (a), (b) and left panel of (f), two‐way ANOVA with major effect between groups in (c)–(e) and Chi square test in (f) right panel. All data are presented as the mean ± SEM. *p < 0.05; **p < 0.01; ***p < 0.001; ****p < 0.0001.

The balance between excitation and inhibition is tightly regulated and essential to maintain neural network dynamics (Froemke, 2015). We ask if the hyperactivity of BLA neurons in basal state of AD is caused by an imbalance of excitation and inhibition. To evaluate the function of the BLA neural network, we measured spontaneous excitatory postsynaptic potential currents (sEPSCs) and spontaneous inhibitory postsynaptic currents (sIPSCs), and observed that within the BLA of AD mice, the amplitude and frequency of sEPSCs significantly increased, while the amplitude and frequency of sIPSCs significantly decreased (Figure 2a,b). We further assessed the intrinsic excitability of BLA neurons with whole‐cell patch clamp techniques. We observed that AD pyramidal neurons exhibited higher resting membrane potential (RMP) and burst more action potential than WT neurons upon either depolarizing currents injection or cell‐attached configuration (Figure 2c; Figure S3a,b). The results suggested that aberrant modulation of excitation and inhibition in BLA local circuits occurred in early‐stage AD, which may lead to an imbalance in neural network dynamics. We then analyzed the input–output curve of evoked‐EPSCs (eEPSCs) and observed a reduced synaptic current in AD neurons (Figure 2d).

Somatostatin (SOM) and parvalbumin (PV) interneurons are two major subtypes of inhibitory neurons in the BLA that target the distal dendritic and perisomatic regions of postsynaptic principal neurons, respectively, to exert their distinct inhibitory effects on principal neurons (Jiang et al., 2021). We tested the intrinsic properties of PV interneurons and SOM interneurons by crossing PV‐Cre/SOM‐Cre with Ai9 mice and observed a significant decrease in RMP and action potential firing rate of amygdala PV interneurons in APP/PS1 mice compared to the WT group (Figure 2e; Figure S3c), but no obvious change was detected in SOM interneurons (Figure S3d,e). To test how abnormal PV interneurons affect neighboring pyramidal neurons, we analyzed the paired pulse ratio (PPR) in pyramidal neurons by activating channelrhodopsin‐2 (ChR2) expressed in PV interneurons. We found that although the connectivity of PV interneurons to pyramidal neurons was not affected between the two groups, the PPR was significantly increased in APP/PS1 mice compared with control mice (Figure 2f), suggesting that less inhibitory neurotransmitter (GABA) was released from PV interneurons in the AD group. No change was observed in SOM interneurons (Figure S3f). All these results suggest that the imbalance of excitation and inhibition caused by pyramidal neurons and PV interneurons may contribute to the high firing rate of BLA principal neurons in an early‐stage AD mouse model.

### 2.3 BLA 5‐HT receptors are abnormally upregulated in early‐stage AD

**FIGURE 3:**
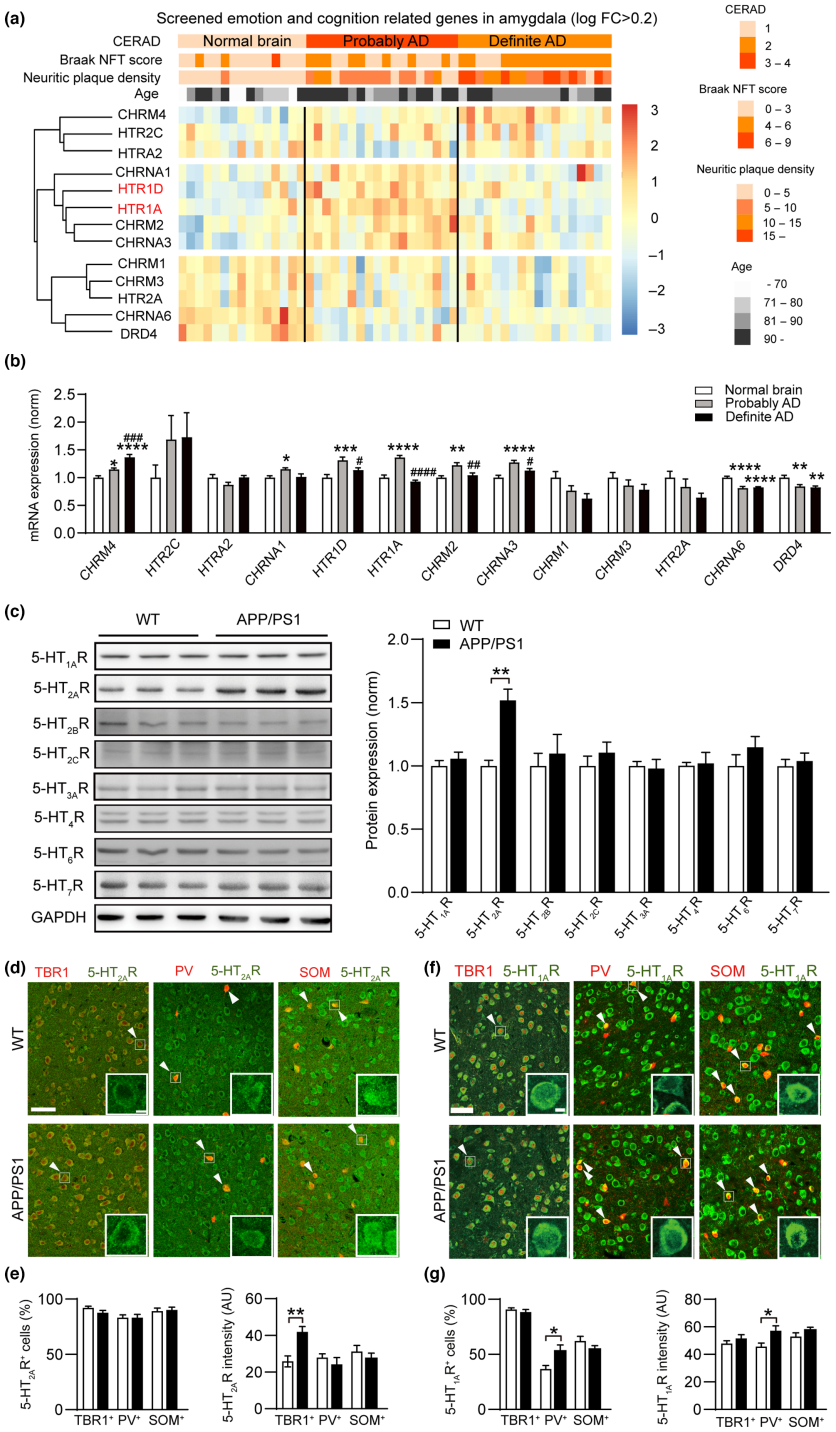
Serotonin receptors are abnormally upregulated during early‐stage AD in the BLA. (a) A hierarchically clustered heatmap showed expression patterns of 13 emotion‐related genes in the human amygdala. (b) The mRNA expression levels of emotion‐related genes in the human amygdala (*n* = 11–18 brain samples for each group). (c) Expression levels of 5‐HT_1A_R, 5‐HT_2A_R, 5‐HT_2B_R, 5‐HT_2C_R, 5‐HT_3A_R, 5‐HT_4_R, 5‐HT_6_R and 5‐HT_7_R proteins in the amygdala from PW10 WT and APP/PS1 mice (*n* = 3 animals for each group). (d, e) Representative images and summary data of 5‐HT_2A_R immunofluorescence co‐localized with different types of neurons in WT and APP/PS1 mice (*n* = 4 to 5 mice for each group). The white arrowheads denote co‐labeled 5‐HT_2A_R^+^/TBR1^+^, 5‐HT_2A_R^+^/PV^+^ or 5‐HT_2A_R^+^/SOM^+^ cells, and the white squares denote the enlarged view. Scale bar, 50 μm; 5 μm for magnified images. (f, g) Representative images and summary data of 5‐HT_1A_R immunofluorescence co‐localized with different types of neurons in WT and APP/PS1 mice (*n* = 6 mice for each group). The white arrowheads denote co‐labeled 5‐HT_1A_R^+^/TBR1^+^, 5‐HT_1A_R^+^/PV^+^ or 5‐HT_1A_R^+^/SOM^+^ cells, and the white squares denote the enlarged view. Scale bar, 50 μm; 5 μm for magnified images. Statistical significance was assessed by unpaired Student's *t* test in (c), (e), and (g), one‐way ANOVA with post hoc comparisons (Tukey test) between groups in (b). All data are presented as the mean ± SEM. * means the difference between normal brain and probably AD brain or normal brain and definite AD brain, # means the difference between probably AD brain and definite AD brain. *p < 0.05; **p < 0.01; ****p < 0.0001; ^#^ *p* < 0.05; ^##^ *p* < 0.01; ^###^ *p* < 0.001; ^####^ *p* < 0.0001.

To explore the molecular mechanisms underlying the abnormal emotion behaviors during the early stage of AD, we profiled the transcriptomes of the amygdala from the postmortem human brain (data come from GSE84422). The samples were divided into normal brain, probable AD brain, and definite AD brain according to the neuropathological traits (Consortium to Establish a Registry for Alzheimer's Disease, CERAD) to show the progression of AD. A hierarchically clustered heatmap showed different expression patterns of 13 key emotion‐related genes in the three groups. Among these genes, we found that in addition to cholinergic receptors, 5‐HT receptors were enriched specifically within amygdala in the probable AD brain rather than in the other groups (Figure 3a,b), suggesting a possible involvement of amygdala 5‐HT receptors in AD.

Accumulating evidence indicates that 5‐HT receptors play important roles in AD and serve as potential therapeutic targets (Geldenhuys & Van der Schyf, 2011; Ramirez et al., 2014; Wang et al., 2023). Considering the remarkably altered 5‐HT receptors in the transcriptome profiles and their potential functions modulating the emotional state, we further precisely detected the expression of related proteins in the amygdala of AD mice. Western blotting data revealed that 5‐HT_2A_ receptor (5‐HT_2A_R) was upregulated significantly in APP/PS1 mice (Figure 3c). We characterized the expression profile of 5‐HT_2A_R in glutamatergic neurons by co‐staining 5‐HT_2A_R with TBR1, the marker of glutamatergic neurons (Gouty‐Colomer et al., 2016), and in GABAergic neurons by using PV‐Cre::Ai9 and SOM‐Cre::Ai9 mice for labeling PV and SOM interneurons. We found that the expression of 5‐HT_2A_R was dramatically upregulated in glutamatergic neurons but not in GABAergic neurons in 2‐month‐old AD model (Figure 3d,e). Preclinical evidence has supported the utility of 5‐HT_1A_R antagonists for treating cognitive impairments associated with AD, which is likely due to facilitation of glutamatergic transmission by removal of the inhibitory effects of 5‐HT (Dijk et al., 1995; Schechter et al., 2005). We further characterized the expression profile of 5‐HT_1A_R in the amygdala. The staining data revealed that 5‐HT_1A_R was highly co‐expressed with glutamatergic neurons and expressed at low level in GABAergic interneurons. Meanwhile, we observed more 5‐HT_1A_R^+^PV^+^ neurons and increased expression of 5‐HT_1A_R in PV interneurons in the BLA of APP/PS1 mice, indicating upregulated 5‐HT_1A_R levels in PV interneurons in early‐stage AD (Figure 3f,g). Furthermore, we validated that the 5‐HT neurons in the dorsal raphe nucleus can project simultaneously to the PV interneurons and pyramidal neurons in the amygdala (Figure S4). To measure the relative level of 5‐HT in the amygdala, we took advantage of the GRAB_5‐HT_, GPCR‐activation‐based (GRAB) sensor for detecting 5‐HT (Deng et al., 2024; Wan et al., 2021), with fiber photometry. First, we used the paroxetine, a selective serotonin transporter inhibitor (SSRI), to confirm the sensitivity of 5‐HT sensor. We observed that paroxetine‐elicited increase in fluorescence was up to 12.59% change in fluorescence (Δ*F*/*F*_0_). We did not see obvious difference between WT and APP/PS1 mice either in home cage or under EPM conditions (Figure S5). The results reveal higher expression levels of two 5‐HT receptors in excitatory and inhibitory neurons of the amygdala in an early‐stage AD mouse model: the 5‐HT_2A_R receptor is upregulated in glutamatergic neurons, while 5‐HT_1A_R is upregulated in PV interneurons.

### 2.4 Serotonin receptors synergistically regulate neuronal activity and emotional behaviors in early‐stage AD

Although both 5‐HT_2A_R and 5‐HT_1A_R belong to the family of G protein‐coupled receptors, they play different roles in cellular activity. 5‐HT_2A_R couples positively to phospholipase C, which leads to increased accumulation of intracellular Ca^2+^ and inositol phosphates (Boess & Martin, 1994). In contrast, 5‐HT_1A_R is activated to reduce intracellular concentrations of cAMP, by which K^+^ channels are open and Ca^2+^ channels are closed to inhibit neuronal firing (Haleem, 2019). To clarify whether these two subtypes of 5‐HT receptors participate in aberrant amygdala function in AD condition, we selectively knocked down 5‐HT_2A_R in pyramidal neurons with AAV‐CaMKII‐sh5‐HT_2A_R or 5‐HT_1A_R in PV interneurons with AAV‐DIO‐sh5‐HT_1A_R in PV‐Cre mice at PW7 and examined animal emotional performance as well as neuronal excitability at PW10 (Figure S6; Figure S7a,b). No obvious change in OFT or EPM was observed in AD mice after downregulation of either 5‐HT receptor (Figure S7c,d). Furthermore, electrophysiological data showed that knockdown of 5‐HT_2A_R or 5‐HT_1A_R in WT mice reduced firing frequency compared with the control group, and impaired synaptic transmission occurred in sh5‐HT_2A_R‐injected mice (Figure S7e,f). We further evaluated neural network function in the amygdala and observed a reduction in sEPSCs after knocking down 5‐HT_2A_R or 5‐HT_1A_R in the WT group, while the sIPSCs were increased in sh5‐HT_2A_R mice (Figure S7g,h). However, none of the electrophysiological properties were affected by sh5‐HT_2A_R or sh5‐HT_1A_R in AD mice. Based on these results, it appears that knockdown of 5‐HT_2A_R in pyramidal neurons or 5‐HT_1A_R in PV interneurons alone fails to restore abnormal neuronal electrophysiological characteristics and behaviors during early‐stage AD.

**FIGURE 4:**
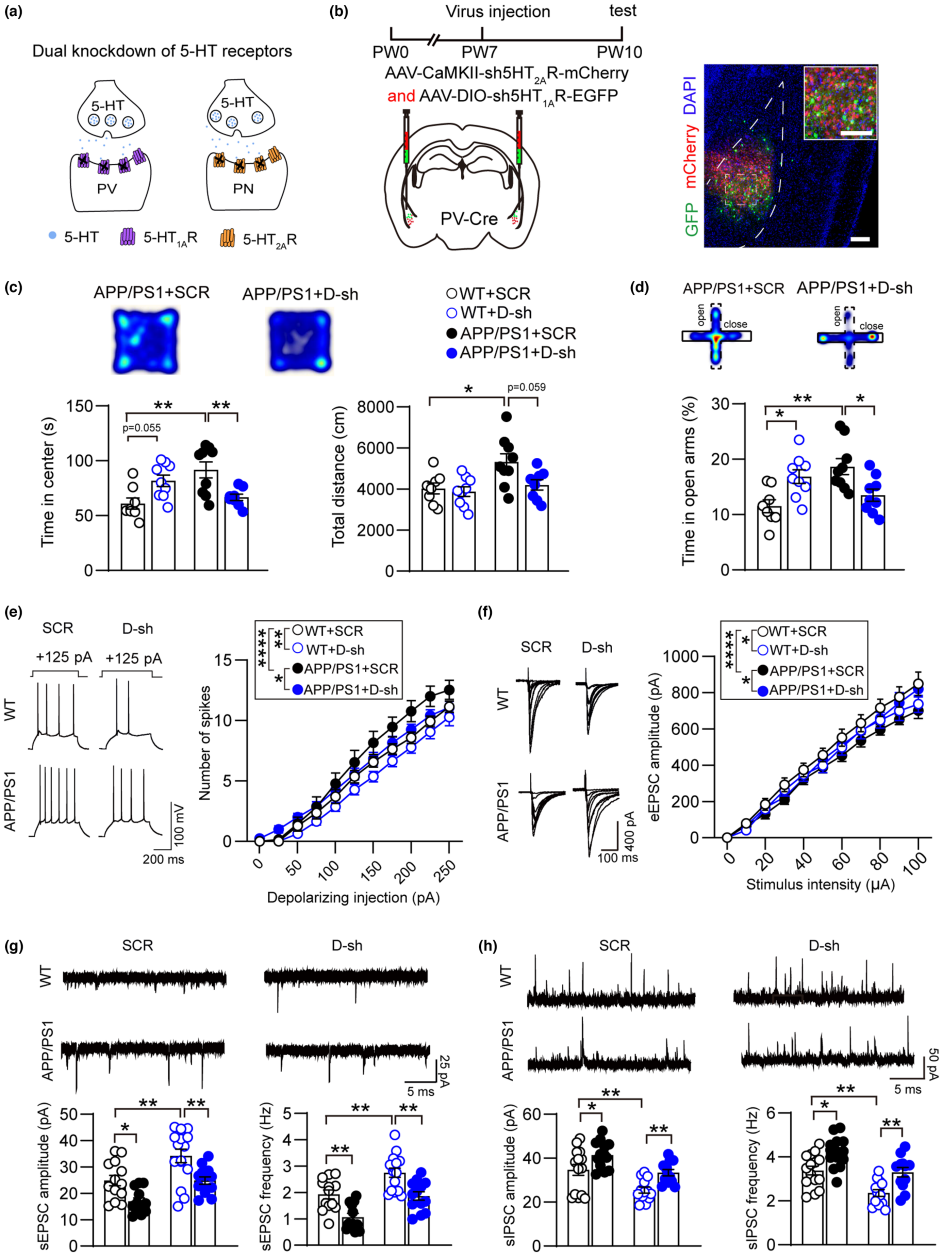
Double knockdown of serotonin receptors rescues mood defeats in APP/PS1 mice. (a) The schematic illustrates combined knockdown 5‐HT_2A_R in pyramidal neuron and 5‐HT_1A_R in PV interneuron. (b) Left panel showed experimental scheme of virus injection in BLA and data analysis. Right panel showed histologically verified placements of viral injections in BLA. Scale bars, 200 μm; 125 μm for magnified images. (c) Knocking down 5‐HT_2A_R in pyramidal neurons and 5‐HT_1A_R in PV interneurons reduce the central time and total distance of APP/PS1 mice in OFT (*n* = 8–10 animals for each group). (d) Knocking down 5‐HT_2A_R in pyramidal neurons and 5‐HT_1A_R in PV interneurons reduce the time in open arms of APP/PS1 mice in EPM (*n* = 8–10 animals for each group). (e) Representative traces for action potential firing of WT + SCR‐shRNA, WT + D‐shRNA, APP/PS1 + SCR‐shRNA and APP/PS1 + D‐shRNA mice and statistical data for action potential firing recorded in four groups (*n* = 12–14 cells from 3 to 4 mice for each group). (f) Sample traces of eEPSC from WT + SCR‐shRNA, WT + D‐shRNA, APP/PS1 + SCR‐shRNA and APP/PS1 + D‐shRNA mice and summary data for eEPSCs from the four groups (*n* = 12–16 cells from 3 to 4 mice per group). (g) Sample traces of sEPSC from WT + SCR‐shRNA, WT + D‐shRNA, APP/PS1 + SCR‐shRNA and APP/PS1 + D‐shRNA mice and summary data for sEPSCs from the four groups (*n* = 12–15 cells from 3 to 4 mice per group). (h) Sample traces of sIPSC from WT + SCR‐shRNA, WT + D‐shRNA, APP/PS1 + SCR‐shRNA and APP/PS1 + D‐shRNA mice and summary data for sIPSCs from the four groups (*n* = 12–15 cells from 3 to 4 mice per group). Statistical significance was assessed by two‐way repeated measures ANOVA with post hoc comparisons (Tukey test) between groups in (c)–(h). All data are presented as mean ± SEM. *p < 0.05; **p < 0.01; ****p < 0.0001.

We then combined knockdown of 5‐HT_2A_R in pyramidal neurons and 5‐HT_1A_R in PV interneurons by application of double shRNA (referred to as ‘D‐shRNA’) in PW7 AD mice to examine emotion‐related behaviors in early‐stage AD (Figure 4a,b). The double‐knockdown AD mice exhibited decreased time spent in the open arms of the EPM or center area of the OFT (Figure 4c,d), suggesting a restoration in emotional performance. Analysis of neuronal firing frequency in APP/PS1 mice showed a restoration of action potential number to the normal level that was comparable to WT mice (Figure 4e). In addition, eEPSCs, sEPSCs, and sIPSCs were also rescued in double‐knockdown AD mice (Figure 4f–h). These observations indicate that the 5‐HT_1A_R of PV interneurons and the 5‐HT_2A_R of pyramidal neurons synergistically maintain the neurological balance of BLA, and reversing the overexpression of 5‐HT receptors in BLA neurons can rescue abnormal mood state during AD progression.

### 2.5 Long‐term downregulation of BLA 5‐HT receptors ameliorates cognitive deficits in AD

**FIGURE 5:**
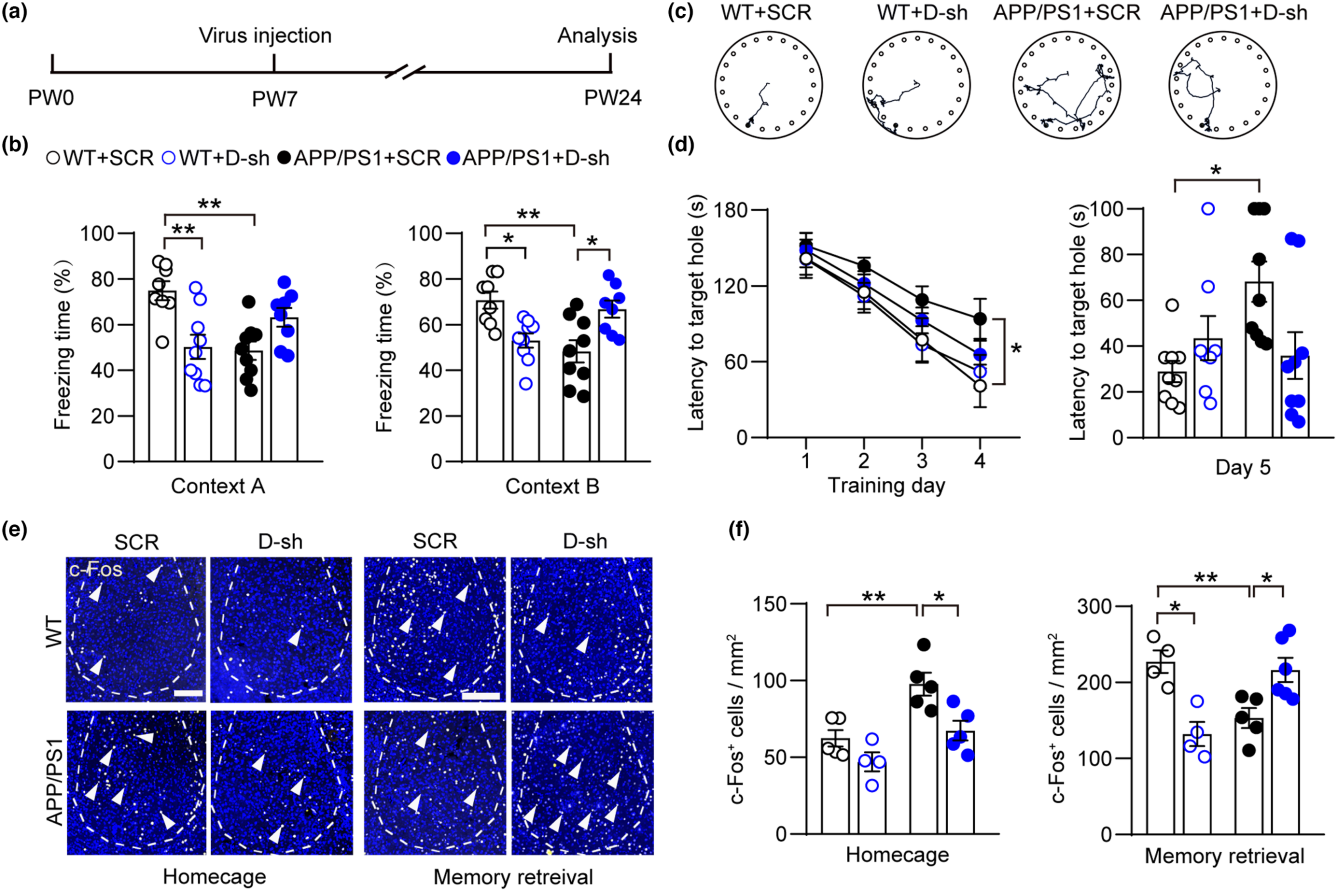
Long term knockdown of dual serotonin receptors in the BLA ameliorates cognitive deficits. (a) Experimental scheme of virus injection in BLA, behavior test and data analysis. (b) Quantification of fear memory after double knockdown of 5‐HT receptors in the BLA of the WT and APP/PS1 mice at PW24 (*n* = 8–9 animals for each group). (c) Representative images for exploring paths of different groups on day 5 in BMT. (d) Quantification of spatial memory after double knockdown of 5‐HT receptors in the BLA of the WT and APP/PS1 groups at PW24 (*n* = 8–10 animals for each group). (e, f) Representative images and summary data of c‐Fos^+^ cells under homecage and memory retrieval in amygdala from WT + SCR‐shRNA, WT + D‐shRNA, APP/PS1 + SCR‐shRNA and APP/PS1 + D‐shRNA mice in PW24 (*n* =  4 to 6 mice for each group). The white arrowheads denote c‐Fos^+^ cells. Scale bars, 200 μm. Statistical significance was assessed by two‐way repeated measures ANOVA with post hoc comparisons (Tukey test) between groups in (b), (d), and (f). All data are presented as mean ± SEM. *p < 0.05; **p < 0.01.

Since prodromal aberrant neuronal activity has been reported as a critical cause of cognitive deficits and the importance of BLA in memory process (Arieli et al., 2022; Nomura et al., 2015), we investigated whether interfering with the abnormal expression of 5‐HT_1A_R and 5‐HT_2A_R in amygdala neurons of early‐age AD mice could protect neurons and maintain normal memory in the later stages (Figure 5a). We examined the cognitive function of mice at PW24 by using FCT and found that WT mice treated with dual knockdown virus in the BLA showed memory deficits, while double shRNA‐treated APP/PS1 mice showed a restored normal freezing time responding to tone‐cue (Figure 5b). We further tested spatial learning and memory (Barnes maze test, BMT) and observed that APP/PS1 mice spent a significantly longer time finding the target hole in BMT, while there was no difference between APP/PS1 mice with long‐term dual 5‐HT receptors knockdown and the control group (Figure 5c,d). These results indicate that long‐term knockdown of 5‐HT receptors ameliorates emotional memory deficits.

We then examined the amygdala neuronal activity in PW24 AD mice and saw a similar upregulated c‐Fos expression compared to young adult AD mice, which manifested as more c‐Fos^+^ neurons in basal state but less activated cells upon environmental stimulation. After manipulating the expression of 5‐HT receptors in APP/PS1 mice, c‐Fos^+^ cells were also restored to normal conditions (Figure 5e,f), which suggests that 5‐HT_1A_R and 5‐HT_2A_R serve as major mediators for amygdala hyperactivity in AD course from early neuropathology to the advanced phase. Our results demonstrate that chronic modulation of neuronal activity by knocking down two 5‐HT receptors in the BLA specifically ameliorated aberrant neuronal properties in AD mice.

### 2.6 Recovery of neuronal activity in BLA enhances resistance to Aβ neurotoxicity

**FIGURE 6:**
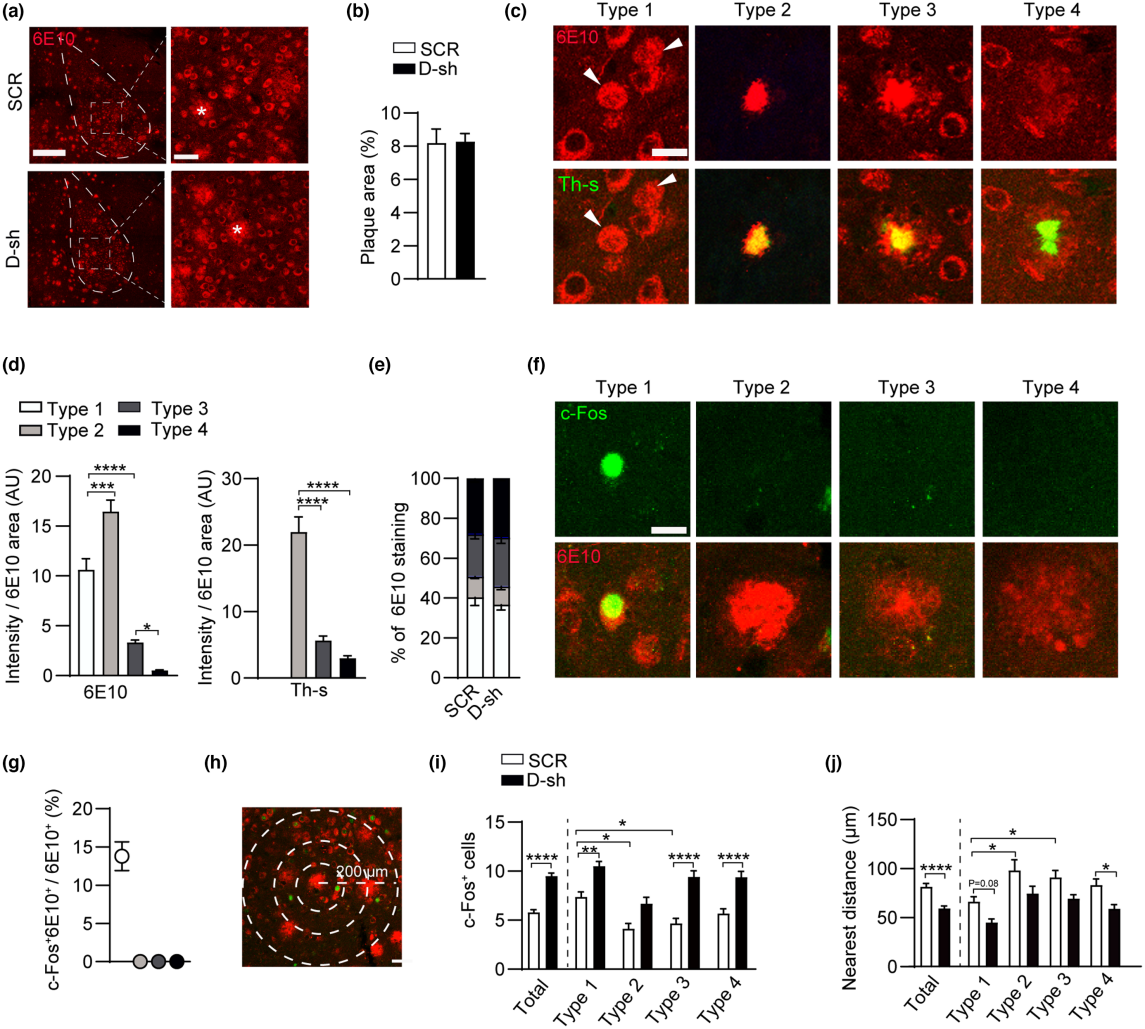
Recovery of neuronal activity of BLA alleviates neurotoxicity of Aβ. (a, b) Representative images and summary data of Aβ plaque in BLA of APP/PS1 + SCR‐shRNA and APP/PS1 + D‐shRNA mice at PW24 (*n* = 9 slices from 3 to 4 mice for each group). The * denote Aβ plaque. Scale bars, 100 μm, 150 μm. (c) Representative images of four types Aβ plaque, 6E10^+^ (red), Thio‐S^+^ (green). White arrowheads indicate type 1 Aβ. Scale bars, 20 μm. (d) The fluorescence intensity for 6E10 or Thio‐S in 6E10^+^ area of four types plaques (*n* = 6–9 plaques for each group). (e) Summary data show percentage of four types Aβ plaque of APP/PS1 + SCR‐shRNA and APP/PS1 + D‐shRNA mice at PW24 (*n* = 9 slice from 3 animals for each group). (f, g) Representative images and summary data for 6E10^+^ (red), c‐Fos^+^ (green) immunofluorescence of four types Aβ plaque (*n* = 3 animals for each group). Scale bars, 5 μm. (h) Diagram of methods for assessing neurotoxicity of Aβ plaque. Scale bars, 50 μm. (i) Quantification of c‐Fos^+^ cells from Aβ plaque to c‐Fos^+^ cell within 200 μm at PW24 APP/PS1 + SCR‐shRNA and APP/PS1 + D‐shRNA mice (*n* = 170–185 plaques from 4 to 5 mice for each group). (j) Quantification of nearest distance from Aβ plaque to c‐Fos^+^ cell within 200 μm in PW24 APP/PS1 + SCR‐shRNA and APP/PS1 + D‐shRNA mice (*n* = 170–185 plaques from 4 to 5 mice for each group). Statistical significance was assessed by two‐tailed unpaired Student's *t* test in left panel of (b), one‐way repeated measures ANOVA with post hoc comparisons (Tukey test) between groups in right panel of (d), and two‐way repeated measures ANOVA with post hoc comparisons (Tukey test) between groups in (e), (i), and (j). All data are presented as mean ± SEM. *p < 0.05; **p < 0.01; ***p < 0.001; ****p < 0.0001.

A progressive accumulation of amyloid β (Aβ) peptides in the brain is one of the hallmarks of AD. Considering that neuronal activity directly promotes the production and secretion of Aβ (Cirrito et al., 2008), we measured the density of Aβ plaques in two groups of mice (the SCR‐shRNA control and D‐shRNA groups) through immunostaining with an Aβ antibody (6E10). No difference was observed in total Aβ plaque density within the BLA between the two groups of APP/PS1 mice (Figure 6a,b), suggesting that manipulating the expression of 5‐HT receptors in the AD model does not alleviate plaque burdens.

As immunostaining with 6E10 and Thio‐S showed diverse shapes of Aβ, we classified it into 4 types based on their morphology: Type 1, full of Aβ in neurons but Thio‐S negative; Type 2, a central dense core surrounded by little fibrillar material; Type 3, a central neuronal core surrounded by a corona of fibrillar material; and Type 4, weakly 6E10 stained deposits with blurred borders and irregular shapes (Figure 6c). Types 2, 3, and 4 were Thio‐S^+^, indicating that the Aβ deposits were composed of aggregated β‐pleated sheet amyloid. Type 2 exhibited a notably higher fluorescence intensity for 6E10, suggesting a predominant enrichment of Aβ (Figure 6d). The relative percentages of these four Aβ types in the BLA of APP/PS1 mice at PW24 were 33.4% for type 1 and 11.9%, 23.8%, and 30.9% for types 2–4, respectively. After downregulation of 5‐HT receptors, the proportions of the four types remained unchanged (Figure 6e). To investigate whether different types of Aβ affect the neuronal response to external stimuli, we stained with 6E10 and c‐Fos after FCT in PW24. We found that 13.8% of type 1 Aβ‐containing neurons showed c‐Fos immunoreactivity, but neurons near type 2–4 plaques scarcely responded to external stimuli (Figure 6f,g), suggesting that aggregation of Aβ plaques impairs neuronal activation. To characterize the role of different types of Aβ in neuronal reactivity, we counted the number of c‐Fos^+^ cells within 200 μm from Aβ and measured the distance from the nearest c‐Fos^+^ cell to the Aβ center (Figure 6h). We found fewer c‐Fos^+^ cells around type 2 Aβ plaques and a longer distance from the nearest c‐Fos^+^ cell to the Aβ center than other types, indicating the abnormal toxicity of type 2 plaques to functional neurons. Compared to the SCR‐shRNA group, the number of c‐Fos^+^ cells around the Aβ was increased, and the nearest distance was shortened in D‐shRNA‐injected PW24 mice (Figure 6i,j). In addition, downregulation of 5‐HT receptors also alleviated Aβ chronic neurotoxicity in PW24 mice except type 2 plaques. The results indicate that although decreasing neuronal excitability by manipulating 5‐HT receptors does not reduce plaque loads, it enables neurons to resist the neurotoxicity of Aβ. Together, these results demonstrate that chronic modulation of 5‐HT receptor signals in the amygdala rescues the neuronal activity that helps to resist neurotoxicity of Aβ and alleviates memory decline.

## 3 DISCUSSION

Our findings clarify that the BLA neuronal dyshomeostasis is involved in the mood and cognitive abnormalities during AD course, which is distinct to cognitive related lesions that occur in medial temporal lobe structures (such as the hippocampus, entorhinal cortex, and perirhinal cortex) during the mid to late stages (Wang et al., 2023). We further uncover the abnormality of 5‐HT receptors in BLA before AD onset, the simultaneous upregulation of 5‐HT_1A_R in PV interneurons and 5‐HT_2A_R in pyramidal neurons, which mediates the imbalance of neuronal excitation and inhibition, as well as emotional and cognitive deficits during the course of AD. Synergistically downregulating the expression of two 5‐HT receptors restores neuronal homeostasis that enables these neurons to tolerate the neurotoxicity of Aβ and hence alleviates cognitive deficits in AD (Graphical abstract).

Early clinical studies have found that NPSs are common, with 50% of AD patients having at least one NPS (Peters et al., 2015). Accumulating studies indicate that emotional events often attain a privileged status in memory, which is supported by epidemiological data that early adverse emotional symptoms exacerbate impairments of cognitive function in dementia (Snyder et al., 2018; Srivareerat et al., 2009). The depressed AD patients are more cognitively impaired and disabled than the non‐depressed AD patients (Rovner et al., 1989). More rapid decline of cognition over time is related to increasing levels of apathy (Johansson et al., 2022). Furthermore, cognitive preservation is associated with attaining optimal benefits from treatment against NPSs in a longitudinal treatment course of patients with AD (Nagata et al., 2018). In conclusion, patients with NPSs in early AD course develop dementia symptoms earlier.

As a limbic region, the BLA is critically involved in the processing of mood information and has widespread reciprocal connections with many cognitive regions, such as memory‐related regions (hippocampus) and perceptual pathways (primary visual cortex and inferior temporal cortex) (Choi et al., 2021; Tye et al., 2011). This connectivity enables the BLA to receive rich sensory information that further strengthens the neural representation of emotional stimuli via feedback to sensory pathways and memory regions. Damage to the BLA induces a rare phenomenon in which noxious stimuli remain detected and discriminated against but are devoid of perceived unpleasantness to motivate avoidance that eventually compromises cognitive function (Hebben et al., 1985). Several studies have reported that lesions in the amygdala occur in AD or the prodromal stages of AD (Gonzalez‐Rodriguez et al., 2023; Markesbery et al., 2006; Poulin et al., 2011; Wachinger et al., 2016), and they are related to certain preclinical symptoms, such as emotional dysfunctions (Poulin et al., 2011). Furthermore, its functional radiomics features might be early biomarkers to represent the mood disorders during the MCI course (Feng et al., 2021). Our study finds that the dysfunction of BLA neurons plays an essential role in NPSs and cognitive impairment during the pathogenesis of AD.

The impairment of cognitive functions in AD is thought to largely result from a reduction in neuronal and synaptic activities according to both laboratory and clinical studies (Roy et al., 2016). Neuronal hyperactivity in the neocortex and hippocampus has recently emerged as a particularly noxious process for the finely tuned circuit functions that underlie cognition (Palop & Mucke, 2016; Selkoe, 2019). There is an opinion that a gradual neuronal “silencing” that occurs after neuronal overexcitation may be the prelude to neurodegeneration (Busche & Konnerth, 2015). Our previous studies indicate that calcium dyshomeostasis in AD leads to abnormally high neuronal activity (Sun et al., 2014; Zhang et al., 2010; Zou et al., 2022). In this study, we find that BLA pyramidal neurons exhibit aberrantly high activity and insensitivity to external stimuli during the AD course. Once the hyperactivity of pyramidal neurons under the baseline state is inhibited, impaired synaptic transmission, abnormal emotion behaviors, and memory deficits in AD mice are ameliorated. Therefore, we speculate that the neuronal dysfunction of BLA‐mediated NPSs and memory impairment may also be associated with abnormal ion channels in neurons. As we know, excitation/inhibition (E/I) imbalance is a potential cause of neuronal network malfunctioning in AD, further contributing to cognitive dysfunction. Previous studies focusing on the glutamatergic system indicate that hyperactivity is initiated by the suppression of glutamate reuptake (Zott et al., 2019). Furthermore, growing evidence also shows dysfunction of GABAergic neurons and GABA receptors leads to E/I imbalance (Bi et al., 2020), such as downregulation of the voltage‐gated Nav1.1 channel, which reduces PV interneuron excitability and causes cortical network hyperactivity (Verret et al., 2012). Recent study indicates that abnormal 5‐HTergic signaling in the hippocampal CA1 region of 4‐ to 5‐month‐old AD model mice led to hyperexcitability of pyramidal neurons (Wang et al., 2023). In contrast to the previous studies, our results indicate that BLA neuronal hyperactivity is attributed to the imbalance of E/I in PV interneurons and pyramidal neurons mediated by 5‐HT receptors, which further contributed to emotional and cognitive abnormalities in the AD process. Our results indicate that dual mechanism of E/I imbalance within the BLA neurons is critical for AD pathogenesis.

A growing number of studies supported 5‐HT receptors as the targets for improving cognitive dysfunction in AD, including 5‐HT_1B_R, 5‐HT_1D_R, and 5‐HT_6_R (Rodríguez et al., 2012). Solas et al. (2021) found that 5‐HT_7_R may involve in NPSs of AD. Among the various 5‐HT receptors, 5‐HT_1A_R is relevant to AD as they are highly expressed in hippocampus and are known to be involved in the regulation of memory processes (Verdurand & Zimmer, 2017). Preclinical evidence has supported the utility of 5‐HT_1A_R antagonists for treating cognitive impairments associated with AD, which is likely due to facilitation of glutamatergic transmission by removal of the inhibitory effects of 5‐HT (Dijk et al., 1995; Schechter et al., 2005). However, there were significant improvements for symptoms of AD patients after the start of administration of 5‐HT_1A_R agonists (Sato et al., 2007). A silent mutation in 5‐HT_2A_R may be a risk factor for NPSs in the course of AD (Tang et al., 2017). Furthermore, clinically first‐line antiallergic drug desloratadine (DLT) functioned as a selective antagonist of 5‐HT_2A_R and efficiently ameliorated pathology of APP/PS1 mice (Lu et al., 2021). In this study, we uncover the abnormality of 5‐HT receptors in BLA before AD onset and the simultaneous upregulation of 5‐HT_1A_R in PV interneurons and 5‐HT_2A_R in pyramidal neurons, which mediates excitation and inhibition imbalance, and further causes cognitive deficits during the course of AD. Our results indicate that excitation and inhibition interplay mediated by 5‐HT receptors within the BLA is critical for AD pathogenesis.

The amyloid‐β peptide is involved in almost all known forms of familial AD. The neurotoxicity of endogenous Aβ participates in maintaining persistent neuronal hyperactivity (Kamenetz et al., 2003). As a negative feedback mechanism, higher neuronal activity conversely serves as an important contributor to Aβ peptide secretion in various experimental settings (Bero et al., 2011; Yuan & Grutzendler, 2016). It is noticeable that maintaining basal state BLA neuronal homeostasis by downregulating 5‐HT receptors enhances the tolerance of Aβ to neurotoxicity but does not alter amyloid plaque burdens. In contrast to the morphological classification of amyloid plaques by using Thio‐S staining and investigation of toxicity via neuronal structure in a previous study (Bussière et al., 2004), we used 6E10 and Thio‐S to categorize the plaques and quantify the related parameters of c‐Fos^+^ cells. It seems that type 2 plaques serve as the most toxic type to impair function of neurons, which is worthy of further investigation.

In summary, we identify the BLA as an essential dysfunctional core, exhibiting aberrant neuronal homeostasis in the basal state and insensitivity to external stimuli in the AD course, from prodromal NPSs to later memory decline. Since behavioral and personality changes are among the most prominent symptoms in the early stage of frontotemporal dementia (Lansdall et al., 2017), vascular dementia (Anor et al., 2017), and Parkinson's disease (Weintraub et al., 2022), our findings would lead to a better understanding of the prodromal mechanism for preclinical neuropsychiatric symptoms and later deficits, which may provide potential valuable therapies for the treatment of neurodegenerative diseases.

## 4 METHODS AND MATERIALS

### 4.1 Animals

APP/PS1 (*APP* ^KM670/671NL^; *PS1* ^L166P^) mice were kindly provided by original lab. *Fos* ^2A‐iCreER^ (TRAP2) mice were purchased from the Jackson Laboratory (stock no. 030323). PV‐IRES‐Cre and SOM‐IRES‐Cre mice were the generous gifts of Prof. Tian‐Le Xu. All the mice were backcrossed to the S129 strains. Both male and female mice were used in all experiments, with a sex ratio of approximately 1:1. Mice were housed with 12‐h light–dark cycle and allowed to receive food and water ad libitum. Animal care and use conformed in accordance with the US Nation Institutes of Health Guide for the Care and Use of Laboratory Animals under an Institutional Animal Care and Use Committee‐approved protocol (Approval number A2022095) and in facilities approved by the Experimental Animal Care Evaluation and Accreditation Association of Shanghai Jiao Tong University School of Medicine.

### 4.2 Behavior test

All animals underwent handling and had 3 days to habituate behavioral room before any tests began. Mice were placed in the experimental room at least 30 min before test for habituation. Animals for behavioral tests were randomly selected to experimental groups, and data analysis was performed blinded to the animal genotypes. All behavioral tests were conducted daily in the light cycle between 13:00  and 17:00  and were recorded with EthoVision software (Noldus Information Technology, Leesburg, VA).

#### 4.2.1 Elevated plus maze (EPM)

Animals were placed in the central area of the apparatus with their head facing an enclosed arm and allowed to explore freely for 5 min. The amount of time that mice spent in the open arms was calculated.

#### 4.2.2 Open field test (OFT)

The arena (40 cm × 40 cm × 40 cm) was used, and mice were placed in the central area and recorded for 20 min. The amount of time and distance that mice spent in the center region of the open field were calculated, and total distance was also counted.

#### 4.2.3 Fear conditioning test (FCT)

The experiment was performed by using the Ugo Basile Fear Conditioning System (UGO BASILE srl). The conditioning chambers (17 cm × 17 cm × 25 cm) equipped with stainless steel shocking grids were connected to a precision feedback current‐regulated shocker. On the day before the fear conditioning training, mice were placed in the chamber with wallpapers (context A) to explore freely for 20 min. On the training day, mice were also placed in context A to explore freely for 3 min firstly, then a pure tone (CS) was played for 28 s, followed by an electric foot shock (US, 0.75 mA, 2‐s duration) through the floor grid. One minute later, paired CS‐US repeated. Conditioned mice were returned to their home cages 30 s after the end of the last tone, and the floor and walls of the cage were cleaned with 75% ethanol for each mouse. One day after conditioning, mice were placed in context A to test the freezing time (contextual fear memory). And animals received the CS‐alone presentations in a test chamber, which had non‐shocking floor with different wallpapers and was washed with 4% acetic acid solution between the tests for individual mice (context B). In detail, the mice explored freely for 3 min and then the tone was played continually for 1 min in context B. We analyzed the freezing behavior during tone presentations. The freezing time in context B refers to auditory fear memory. Mice behaviors were recorded by digital video cameras mounted above the conditioning chamber.

#### 4.2.4 Barnes maze test (BMT)

The BMT was conducted using a circular platform that is 100 cm in diameter and has 20 evenly spaced holes (4 cm diameter, 2 cm away from the edge). Only one hole led to a drop box in which animals could hide. On the first day, mice were allowed to explore for 3 min per trial to find the hiding box. We performed 4 consecutive days of training trials with 2 trials per day. Mice were gently guided to the escape hole if they did not find the hiding box by the end of 3 min. On day 5, the hiding box was removed, and a 100‐second probe trial was performed. Spatial memory abilities were assessed as latency to the hiding box (day 1–day 4) or the escape hole (day 5).

### 4.3 Neuronal tagging

Recombination was induced with tamoxifen. Before tagging, tamoxifen was dissolved at 10 mg/mL in corn oil at 60°C for 30 min, and then it was shake at 37°C for 15 min. All injections were delivered intraperitoneally (i.p.). Mice were transported from the vivarium to an adjacent holding room at least 3 h before the tamoxifen injections to minimize transportation‐induced immediate early gene activity. Activity‐dependent neuronal tagging was induced by a single intraperitoneal injection of tamoxifen (150 mg/kg per mice) 30 min before EPM behavior.

### 4.4 Bioinformatics analysis

GSE84422 dataset was downloaded from GEO (Gene Expression Omnibus, https://ncbi.nlm.mih.gov/geo/) database of NCBI. According to original study (Wang et al., 2016), all the samples were divided into normal brain, probable AD brain, and definite AD brain according to the neuropathological traits (Consortium to Establish a Registry for Alzheimer's disease, CERAD) to show the progression of AD. AD diagnoses were coded as follows: 1 = normal, 2 = definite AD, 3 = probable AD, and 4 = possible AD. According to the diagnostic subgroup of AD disease, the raw data was further analyzed. Preprocessing of the raw probe‐level data in CEL file was conducted through the robust multi‐array average (RMA) algorithm in the Affy package of the R 3.4.3 language, including background correction, quartile data normalization, and conversion into expression measures. For a gene that corresponds to multiple probes, we took the average of the probe values as the expression value. Missing data were imputed with the *k*‐Nearest Neighbor (KNN) approach (*k* = 10). Heat maps of these mood genes were obtained through the heat map package.

### 4.5 Virus constructs

The following viruses were used: AAV‐hSyn‐GCaMP6s was purchased from Shanghai Taitool Bioscience Co. Ltd.; AAV‐DIO‐ChR2(H134R)‐EYFP, AAV‐CaMKIIα‐5‐HT_2A_R‐shRNA‐mCherry (sequence AAAGCTGCAGAATGCCACCAACT) (Kim et al., 2021), AAV2/1‐hSyn‐Cre and AAV‐CaMKIIα‐mCherry were purchased from BrainVTA; AAV‐DIO‐5‐HT_1A_R‐shRNA‐EGFP(sequence AAGAAGATCATCAAGTGCA), AAV‐DIO‐EGFP and AAV‐DIO‐mCherry were purchased from Obio Technology Co. Ltd. AAV‐hSyn‐5HT3.0 was purchased from WZ Biosciences. All viral vectors were stored in aliquots at −80°C until use. The viral titers for injection were more than 10^12^ viral particles per mL.

### 4.6 Stereotaxic surgery

Stereotaxic surgeries were performed as previously described (Wu et al., 2020). Mice were anesthetized and placed in a stereotaxic frame (RWD Instruments, Shenzhen, China). A feedback heater was used to keep mice warm during surgeries. We delivered 300 nL of virus through a pulled‐glass pipette at a slow rate (50 nL/min). Viral injections were targeted into amygdala using coordinates (BLA: AP, −1.50 mm; ML, ±3.30 mm; DV, −4.90 mm; DRN: AP, −4.50 mm; ML, 0 mm; DV, −3.50 mm) based on the Paxinos and Franklin mouse brain atlas (2nd edition). Following infusion, the needle was kept at the injection site for 10 min and then slowly withdrawn. Following injection, the mice were collected on a 37°C warm plate for recovery. Mice were allowed to recover for 15 to 21 days before starting the experiments. At the end of the experiment, mice used for behavioral tests were perfused and checked for injection sites.

### 4.7 Western blotting

Amygdala regions were dissected from WT and APP/PS1 mice at 2–3 months and solubilized at 4°C for 1 h in lysis buffer (1% CHAPS, 137 mM NaCl, 2.7 mM KCl, 4.3 mM Na_2_HPO_4_, 1.4 mM KH_2_PO_4_, 5 mM EDTA, 5 mM EGTA, 1 mM PMSF, 50 mM NaF, 1 mM Na_3_VO_4_, and protease inhibitors, pH 7.2). Lysates were centrifuged at 13,000 *g* for 15 min at 4°C to remove the insoluble deposit. Samples were boiled, run on SDS polyacrylamide gels (9%), and transferred to nitrocellulose membranes. Immunoblotting was performed with the following primary antibodies overnight at 4°C, then incubated with appropriate HRP‐conjugated secondary antibodies diluted in 3% BSA in TBST for 1 h at room temperature. The washed membranes (three times for 10 min each with agitation) were added with Clarity Western ECL and detected with digital imaging equipment (Tanon 5200 Multi). Antibodies for western blotting are asfollows: 5‐HT_1A_R antibody (1:1000, Abcam, ab85615), 5‐HT_2A_R antibody (1:1000, Millipore, MABN1595), 5‐HT_2B_R antibody (1:1000, Abcam, ab194333), 5‐HT_2C_R antibody (1:1000, Abcam, ab37293), 5‐HT_3A_R antibody (1:1000, Alomone Labs, ASR‐031), 5‐HT_4_R antibody (1:1000, Abcam, ab60359), 5‐HT_6_R antibody (1;500; Abcam, ab103016), 5‐HT_7_R antibody (1:1000, Abcam, ab137493), GAPDH (1:5000; Sigma‐Aldrich, G8795).

### 4.8 Brain slice electrophysiology

Brain slices were prepared from 2 to 3 month‐old naïve mice. All experiments were performed blind to genotype. Mice were anesthetized with isoflurane, and brains were dissected quickly and chilled in ice‐cold artificial cerebrospinal fluid (ACSF) containing (in mM): 125 NaCl, 2.5 KCl, 2 CaCl_2_, 1 MgCl_2_, 25 NaHCO_3_, 1.25 NaH_2_PO_4_, and 12.5 glucose (Sigma Aldrich) saturated with 95% O_2_ and 5% CO_2_. Brains coronal brain slices (300 μm thick) were prepared with a vibratome and recovered in ACSF bubbled with 95% O_2_ and 5% CO_2_ at 31°C for 2 h and then maintained at room temperature (22–25°C). The recording pipettes (3–5 MΩ) were filled with a solution. The initial access resistance was 10–30 MΩ, and it was monitored throughout the experiment. Electrophysiological signals were acquired by Axon MultiClamp 700B amplifier, digitized at 10 kHz by a Digidata 1550A D‐A converter, and Bessel filtered at 2 kHz. Data were discarded if the access resistance changed <20% during experiment. Data were analyzed in pCLAMP 10.6 (Molecular Devices), and recordings were made from average of three cells per slice and two to three slices per mouse. Data were analyzed using the Mini‐analysis Program (Synaptosoft) with an amplitude threshold of 5 pA.

Spiking activity of amygdala neurons was measured under current‐clamp mode with an internal solution containing the following (in mM): 145 K‐gluconate, 5 NaCl, 10 HEPES, 2 MgATP, 0.1 Na_3_GTP, 0.2 EGTA, and 1 MgCl_2_ (280–300 mOsm, pH 7.2 with KOH). For cell‐attached, we used a K^+^‐based intracellular solution and recorded in the voltage‐clamp mode.

Spontaneous postsynaptic currents (sPSCs) were recorded at 28°C. The internal solution containing the following (in mM): 132.5 Cs‐gluconate, 17.5 CsCl, 2 MgCl_2_, 0.5 EGTA, 10 HEPES, 4 Mg‐ATP, and 5 QX‐314 chloride (280–300 mOsm, pH 7.2 with CsOH). Each neuron was held in voltage‐clamp mode at −70 mV for sEPSC and at 0 mV for sIPSC recordings (Orefice et al., 2019).

Evoke‐EPSC were separated by interstimulus amplitude of 10–100 μA. Bipolar stimulating electrodes were positioned 100–150 μm away from the recording electrode that was used to record glutamatergic transmission in amygdala. For microcircuit mapping experiments, blue light (475 nm) was delivered by a light‐emitting diode (LED) coupled to a 40× objective to activate ChR2^+^ cells or axonal fibers. Five blue light pulses of 10 mW with 5‐ms duration were applied at a frequency of 1 Hz. ChR2‐expressing interneurons were photostimulated, and non‐fluorescence neurons were recorded. OIPSCs and PPR were averaged across at least 30 light pulses. The internal solution for IPSC recording containing the following (in mM): 140 KCl, 5 NaCl, 2 MgATP, 0.3 NaGTP, 0.1 EGTA, and 10 HEPES (300–310 mOsm, pH 7.2 with KOH).

### 4.9 Immunofluorescence

Mice were anesthetized, and brains were fixed with 4% paraformaldehyde (PFA) solution at 4°C, then mouse brains were sectioned at 40 μm thicknesses using vibrating microtome (Leica, VT1200S). The brain slices were collected and washed three times in PBS to remove embedding agent, then blocked with permeable buffer (0.3% Triton X‐100 in PBS) containing 10% donkey serum for an hour at room temperature and subsequently incubated with primary antibodies in permeable buffer containing 2% donkey serum overnight at 4°C. Then, the samples were washed three times in PBST before incubation with Alexa Fluor secondary antibodies (1:500, Molecular Probes) in the PBS buffer for 2 h at room temperature. Subsequently, brain sections were mounted on slides and then cover‐slipped with DAPI Fluoromount‐G mounting medium. For primary antibodies, we used c‐Fos antibody (1:500, Cell Signaling Technology, 2250), 5‐HT_1A_R antibody (1:500, Millipore, MAB11041), 5‐HT_2A_R antibody (1:200, Millipore, MABN1595), TBR1 antibody (1:200, Millipore, AB2261), PV antibody (1:200, Millipore, MAB1572), Cre antibody (1:200, Cell Signaling Technology, 15,036), serotonin antibody (1:200, Abcam, ab66074), β‐amyloid antibody (1:1000, BioLegend, 803,015). For Thioflavin‐S staining, the brain sections were incubated with primary antibodies and washed in PBST, and followed by 10 min staining with a solution of 0.015% Thio‐S in water.

Images were captured on a Leica SP8 confocal microscope or Olympus VS120 Virtual Microscopy Slide Scanning System. Analysis and quantitative analysis of dual‐labeled neurons in the amygdala were performed with ImageJ software (RRID:SCR_003070). Quantitative analysis of c‐Fos^+^ neurons was performed with Image J software by blinding to the treatment condition. For Aβ neurotoxicity, we counted the c‐Fos^+^ cell number and measured distance from nearest c‐Fos^+^ cell in the concentric circle with a radius of 200 μm centered on Aβ plaque.

### 4.10 Fiber photometry

Fiber photometry experiments were performed as previously described (He et al., 2023). Mice were injected with AAV‐hSyn‐GCaMP6s or AAV‐hSyn‐5HT3.0 into amygdala followed by optic fiber implanted. A 200‐μm‐diameter optical fiber was glued into a short cannula with the fiber tip extended approximately 5 mm out of the cannula. The fiber photometry was conducted by fiber photometry system (Thinker Tech Nanjing BioSicence Inc) 2 weeks later. The analog voltage signals were digitalized at 100 Hz. In vivo recordings were carried out in different behavior, and mice were allowed to explore as described above. All the Ca^2+^ or 5‐HT signals and behavioral videos were synchronized offline with event marks. We calculated the values of fluorescence change (ΔF/F) by calculating (F‐F0)/F0, where F0 was the baseline fluorescence signal. For homecage condition, we analyzed the fluorescence for 10 min, and only Ca^2+^ or 5‐HT signals >2SD were treated as an event. Number of events (>2SD) in 1 min and the percentage of events duration in 1 min were calculated.

### 4.11 Statistical analysis

The results are presented as the mean ± SEM. Statistical differences were determined by Student's *t* test for two‐group comparisons or ANOVA followed by Tukey's test for multiple comparisons among more than two groups. Statistical significance was set at *p* less than 0.05.

## AUTHOR CONTRIBUTIONS

SS and NJX conceived and designed the research study. XRW, XNZ, XG and YZ performed animal behavior. XRW and XNZ carried out electrophysiological recording and quantified the behavioral and calcium data with assistance from XDL, SC and TLX. YBP completed the bioinformatics analysis. XRW, SS and NJX wrote the manuscript. All authors reviewed and edited the manuscript. The authors declare no competing financial interests.

## FUNDING INFORMATION

This research was supported by the Science and Technology Innovation 2030 Major Projects (STI2030‐Major Projects, no. 2021ZD0202801 to N.‐J.X.), Shanghai Outstanding Academic Leaders Program (no. 21XD1401800 to N.‐J.X.), the National Natural Science Foundation of China (no. 32271009 to S.S., no. 32030042 to N.‐J.X.). This study was also supported by the Shanghai Frontiers Science Center of Cellular Homeostasis and Human Diseases. The authors declare no competing financial interests.

## CONFLICT OF INTEREST STATEMENT

The authors declare no competing interests.

## Supporting information


Figures S1–S7.

## Data Availability

The data that support the findings of this study are available from the corresponding author upon reasonable request. Data sharing is not applicable to this article as no new data were created or analyzed in this study. Any additional information required to reanalyze the data reported in this paper is available from the lead contact upon request.
