# Supplementary material for: Amygdala neuronal dyshomeostasis via 5‐HT receptors mediates mood and cognitive defects in Alzheimer's disease
Source: Aging Cell. 2024 May 8;23(8):e14187. doi: 10.1111/acel.14187 (PMC11320345; doi:10.1111/acel.14187)
Supplement: Supplementary file 1 — Figures S1–S7. [file ACEL-23-e14187-s001.pdf]

Amygdala neuronal dyshomeostasis via 5-HT receptors mediates mood and cognitive defects in Alzheimer's disease

Xin-Rong Wu *et al*

Corresponding author:

Suya Sun, sunsuya@shshmu.edu.cn

Nan-Jie Xu, xunanjie@sjtu.edu.cn

This PDF file includes:

Figure S1 to S7

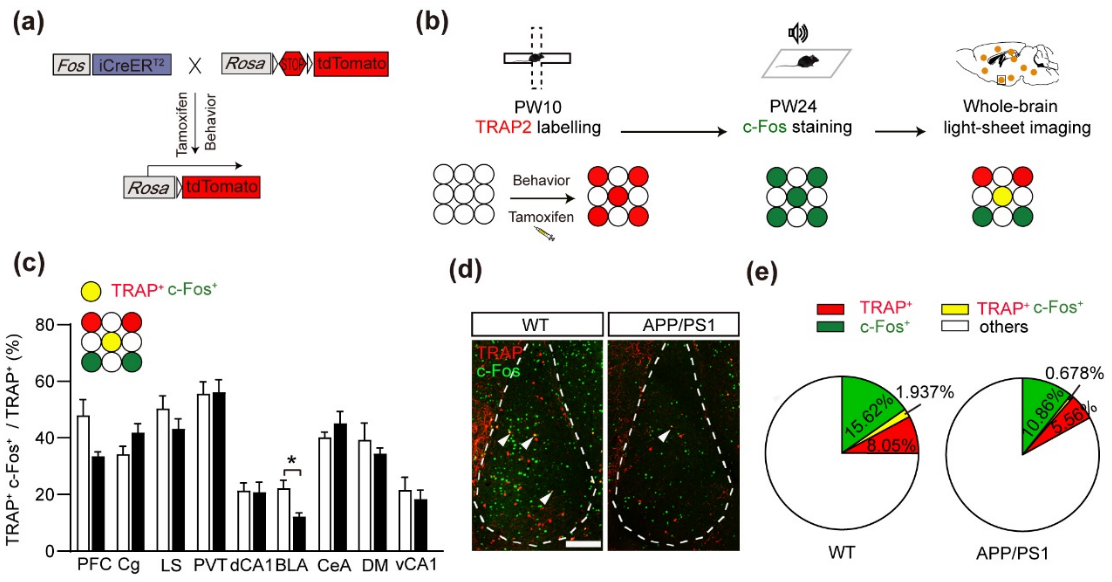

Figure S1. BLA neurons respond aberrantly to emotional and cognitive behaviors during AD pathogenesis.

(a) Illustration of the TRAP strategy used to label active neurons. Mice expressing tamoxifen-inducible Cre recombinase under the control of the Fos promoter (Fos-iCre^{ERT2}) were crossed with Ai9-Tdtomato reporter mice. TdTomato is expressed by behavior and tamoxifen administration to induce Cre-mediated excision of the loxP-flanked PGK neo cassette. (b) The schematics of experimental design. (c) Quantification of TRAP$^+$c-Fos$^+$ / TRAP$^+$ across 9 brain regions in the two groups (n = 3-4 animals for each group). (d) Representative images of TRAP$^+$c-Fos$^+$ cells (yellow immunofluorescence, white arrowhead) in the BLA. Scale bar, 200 µm. (e) The percentage of TRAP$^+$, c-Fos$^+$, and TRAP$^+$ c-Fos$^+$ cells among TBR1$^+$ cells in the BLA of WT and APP/PS1 mice. Statistical significance was assessed by unpaired Student's *t* test in (c). All data are presented as the mean ± SEM. *$p < 0.05$; **$p < 0.01$.

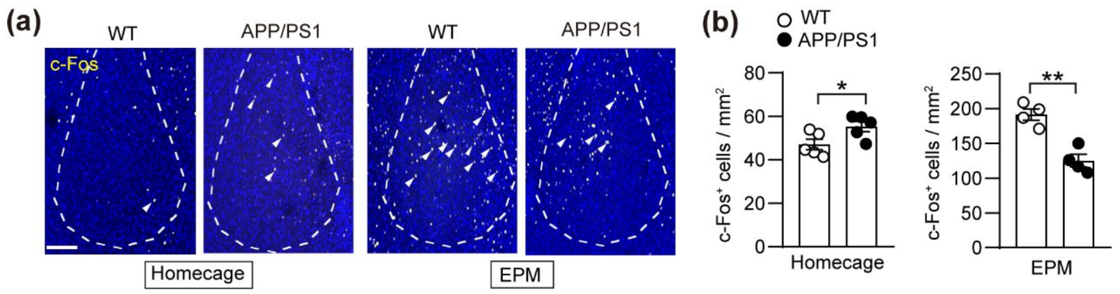

Figure S2. BLA neurons exhibit hyperactivity in the basal state but were blunt to external stimuli in early-stage AD.

(a) Representative images of c-Fos$^+$ cells (yellow, white arrowhead) in the BLA under home cage and EPM conditions. Scale bar, 200 μm. (b) Quantification of c-Fos$^+$ cells in the BLA of WT and APP/PS1 mice (n = 4-5 animals for each group). Statistical significance was assessed by unpaired Student's *t* test in (b). All data are presented as the mean ± SEM. *$p < 0.05$; **$p < 0.01$.

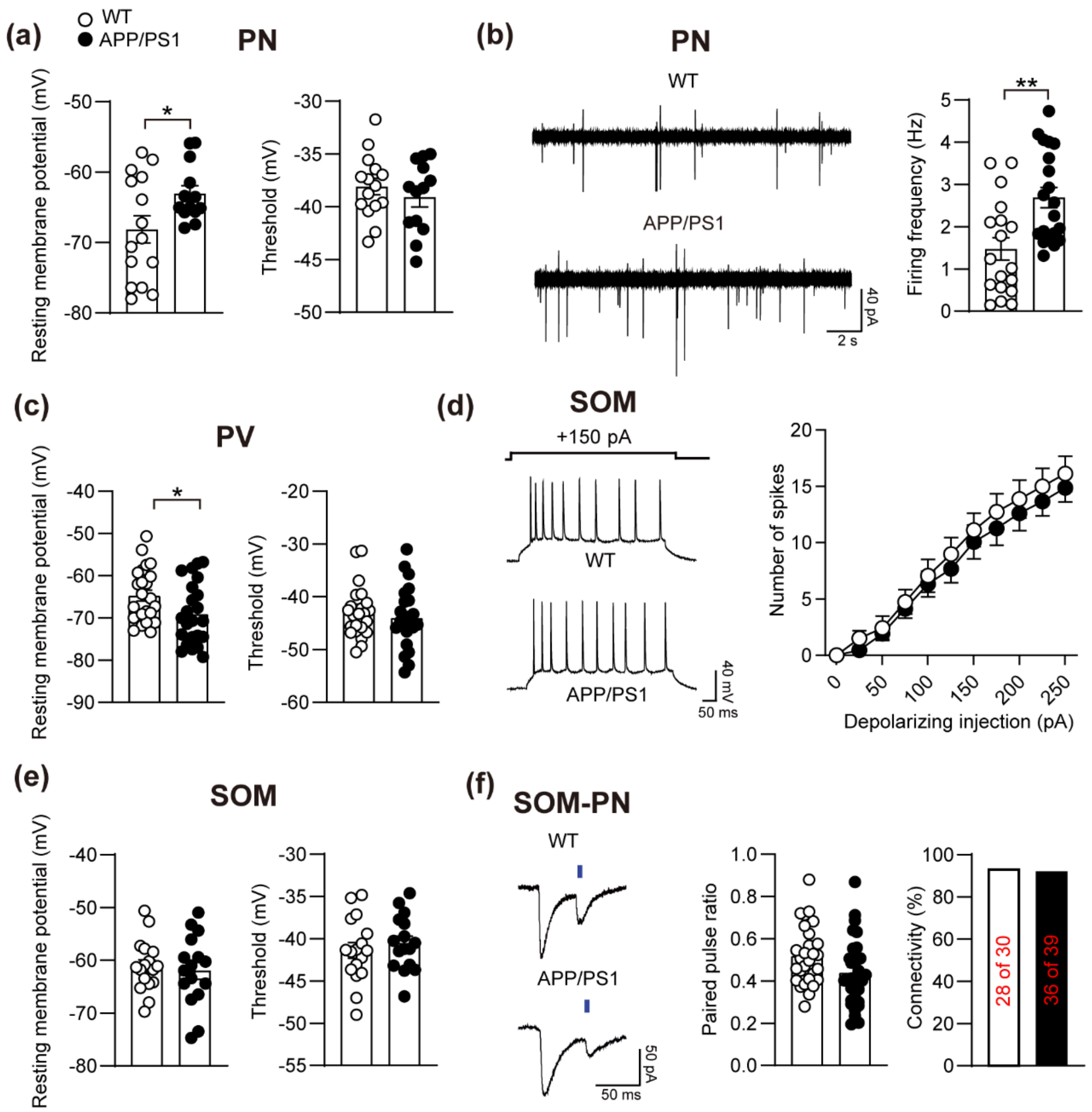

Figure S3. Impaired PV-PN microcircuit in the BLA during early-stage AD.

(a) Quantification of resting membrane potential and threshold in pyramidal neurons between two groups (n = 13-15 cells from 3-4 mice for each group). (b) Sample traces of loose patch and statistical data in the BLA recorded from WT and APP/PS1 mice (n = 18-20 cells from 3-4 mice for each group). (c) Quantification of resting membrane potential and threshold in PV interneurons between two groups (n = 13-15 cells from 4 mice for each group). (d) Representative traces of action potential firing and statistical data from SOM interneurons in two groups (n = 23-24 cells from 3-5 mice

for each group). (e) Quantification of resting membrane potential and threshold in SOM interneurons between two groups (n = 23-24 cells from 3-5 mice for each group). (f) Representative traces and quantification of PPR from SOM to PN in two groups (n = 28-33 cells from 3-5 mice for each group). Significance was assessed by unpaired Student's *t* test in (a), (b), (c), (e) and left panel of (f), two-way ANOVA with major effect between groups in (d), and Chi square test in right panel of (f). All data are presented as mean ± SEM. *$p < 0.05$; **$p < 0.01$.

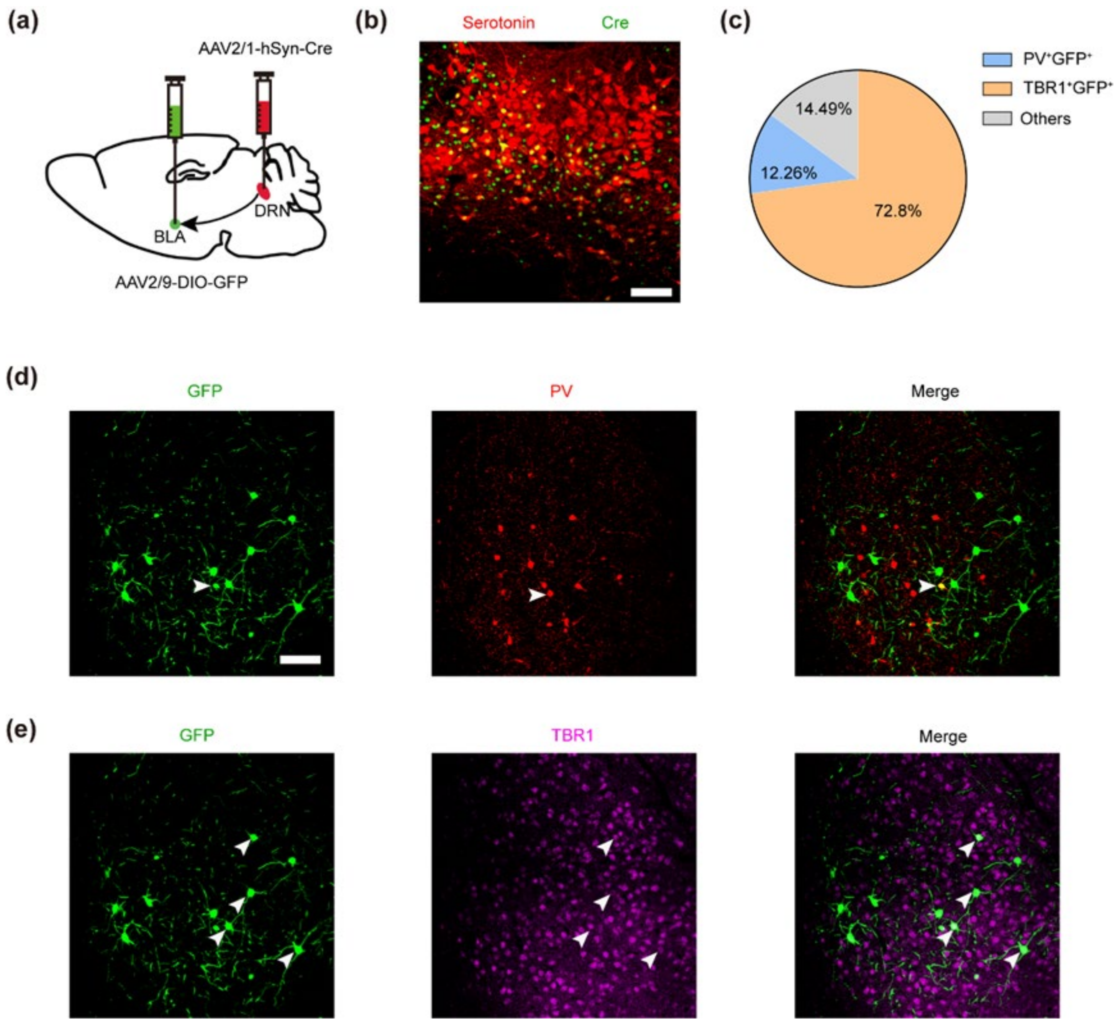

Figure S4 Excitatory projecting and inhibitory projecting coexisted in DRN-BLA.

(a) Schematic of virus injection to 5-HT tracing. (b) Typical image showed immunostaining of cre (green) and serotonin (red) in dorsal raphe nucleus (DRN). scale bar: 100 μm. (c) Quantification of the percentage of $TBR1^+$ GFP^+ cells, PV^+ GFP^+ cells and others. n = 3 animals. (e) Representative immunostaining of GFP (green) and PV (red) in amygdala; scale bar: 100 μm. Arrows indicate GFP^+ PV^+ cells. (d) Representative immunostaining of GFP (green) and TBR1 (violet) in amygdala; scale bar: 100 μm. Arrows indicate GFP^+ $TBR1^+$ cells. All data are presented as mean ± SEM.

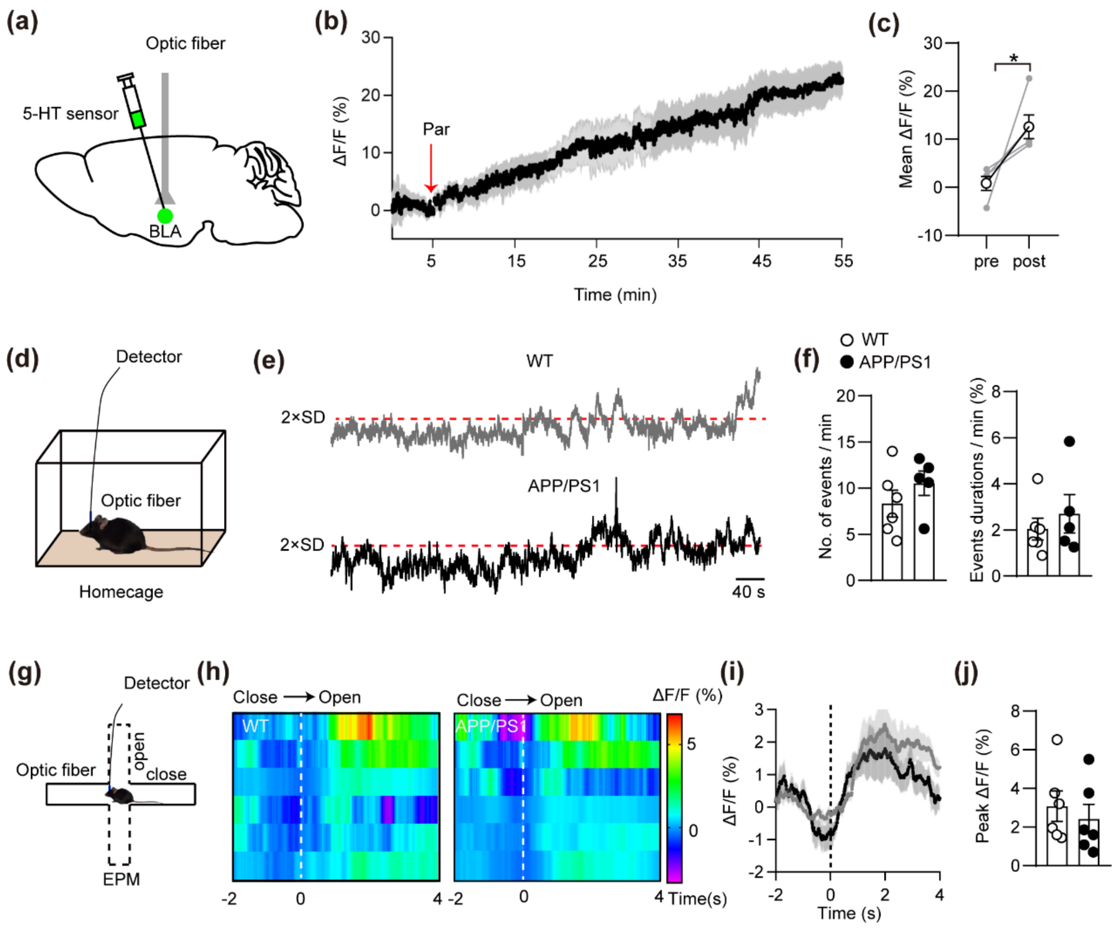

Figure S5. No significant changes are detected for 5-HT neurotransmitter in the BLA during early-stage AD.

(a) Schematic of virus injection to express 5-HT sensor in BLA with the optical fiber implanted above BLA. (b) 5-HT sensor signals from BLA aligned to the moment of the administration of paroxetine (50 mg/kg). (c) Quantification of change in 5-HT sensor signals after administration of paroxetine (50 mg/kg) (n = 4 mice per group). (d) Paradigm of the fiber photometry setup in home cage condition. (e) Representative traces of 5-HT signals in amygdala neurons. (f) Number of events (>2SD) in one minute (left panel) and the percentage of event duration in one minute (right panel) of 5-HT signals (n = 5-6 animals for each group). (g) Paradigm of the fiber photometry setup in

EPM. (h) Heat map illustrating the 5-HT signals (ΔF/F) of BLA neurons when mice moved from closed arms to open arms in EPM in WT mice (left panel) and APP/PS1 mice (right panel). (i) Plot of averaged 5-HT signals during EPM test onto open arms in WT (black) and APP/PS1 mice (gray). Solid line and the shaded regions are the mean ± SEM. Right panel showed the quantification of the peak (ΔF/F) 5-HT signals. (j) Quantification of the peak (ΔF/F) 5-HT signals (n = 6-7 animals for each group). Significance was assessed by paired Student's *t* test in (c), unpaired Student's *t* test in (f) and (j). All data are presented as mean ± SEM.

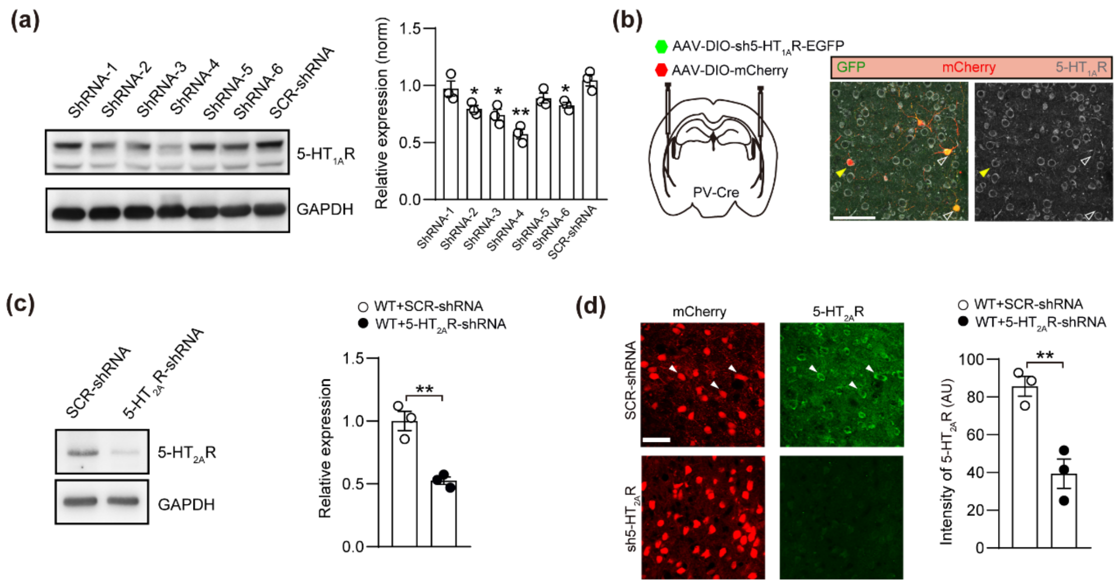

Figure S6. Verification of shRNA and histological verification of viral infection locations in BLA.

(a, b) Western blots and double-immunofluorescence images showed efficient 5-$HT_{1A}R$ knockdown in the amygdala (n = 3 animals for each group). The yellow arrowheads denote $mCherry^{+}sh5\text{-}HT_{1A}R^{+}$ cells, white arrowheads denote $mCherry^{+}$ cells. Scale bar, 100 µm. (c, d) Western blots and double-immunofluorescence images showed efficient 5-$HT_{2A}R$ knockdown in the amygdala (n = 3 animals for each group). The white arrowheads denote $mCherry^{+}sh5\text{-}HT_{2A}R^{+}$ cells. Scale bar, 100 µm. Significance was assessed by two-tailed unpaired Student's *t* test in (c) and (d), one-way repeated measures ANOVA with post hoc comparisons (Turkey test) between groups in (a). All data are presented as mean ± SEM. $*p < 0.05$; $**p < 0.01$.

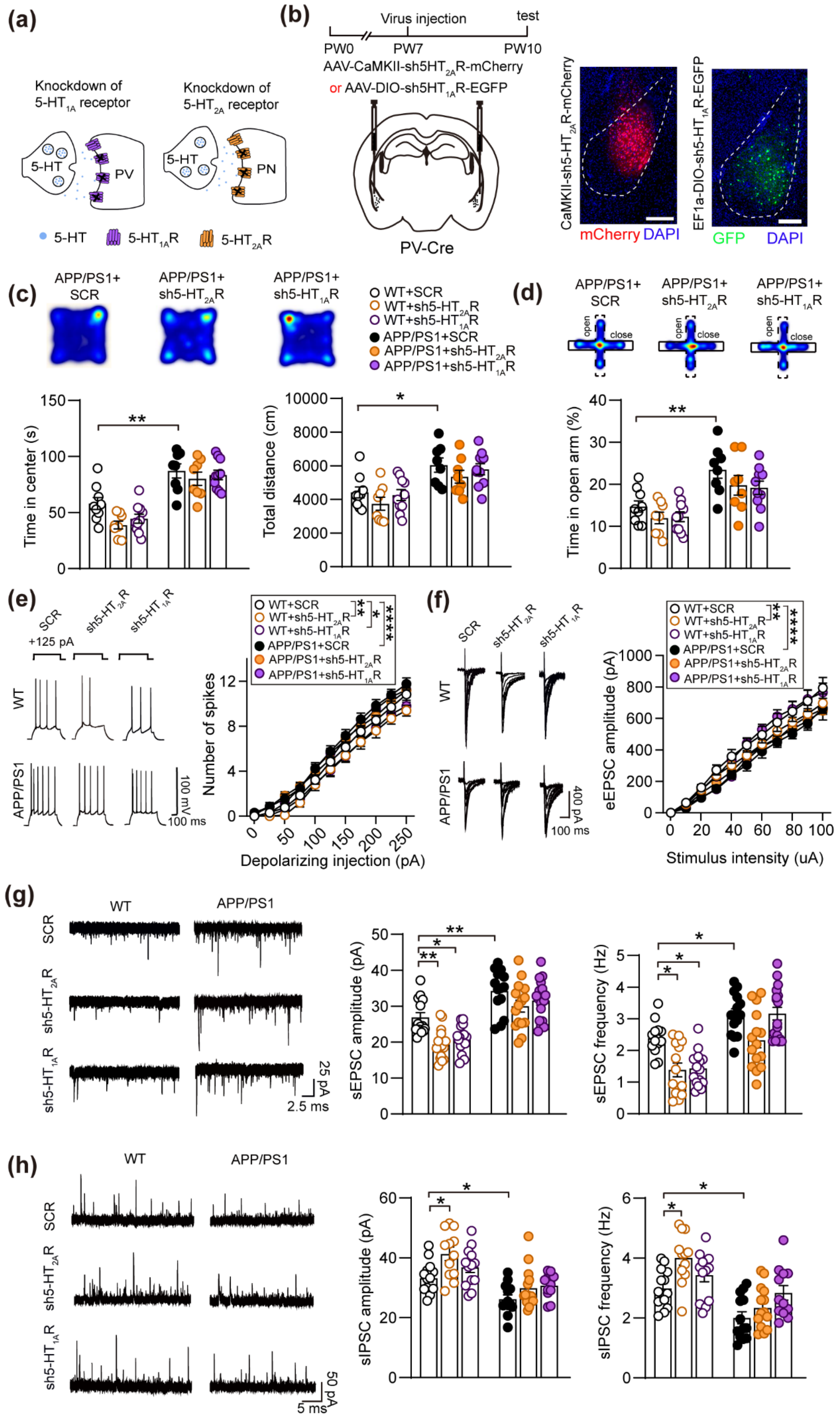

(a)
Knockdown of 5-HT1A receptor
Knockdown of 5-HT2A receptor
5-HT
PV
PN
5-HT
5-HT1AR
5-HT2AR
(b)
Virus injection
test
PW0
PW7
PW10
AAV-CaMKII-sh5HT2AR-mCherry
or AAV-DIO-sh5HT1AR-EGFP
PV-Cre
CaMKII-sh5-HT2AR-mCherry
mCherry DAPI
EF1a-DIO-sh5-HT1AR-EGFP
GFP DAPI
(c)
APP/PS1+ SCR
APP/PS1+ sh5-HT2AR
APP/PS1+ sh5-HT1AR
WT+SCR
WT+sh5-HT2AR
WT+sh5-HT1AR
APP/PS1+SCR
APP/PS1+sh5-HT2AR
APP/PS1+sh5-HT1AR
Time in center (s)
Total distance (cm)
(d)
open
close
Time in open arm (%)
(e)
+125 pA
WT
APP/PS1
100 mV
100 ms
Number of spikes
Depolarizing injection (pA)
(f)
400 pA
100 ms
eEPSC amplitude (pA)
Stimulus intensity (uA)
(g)
sEPSC amplitude (pA)
sEPSC frequency (Hz)
25 pA
2.5 ms
(h)
sIPSC amplitude (pA)
sIPSC frequency (Hz)
50 pA
5 ms

Figure S7. Knockdown 5-HT_{1A}R or 5-HT_{2A}R alone in the BLA do not alleviate the neuronal hyperactivity and emotional defeats.

(a) The schematic illustrates selective knockdown 5-HT_{1A}R in PV interneuron (left) or 5-HT_{2A}R in pyramidal neuron (right). (b) Left panel showed experimental scheme of virus injection in BLA and data analysis. Right panel showed histologically verified placements of viral injections in BLA. Scale bars, 200 μm; 125 μm for magnified images. (c and d) Statistical data for EPM and OFT behaviors from six groups (n = 8-10 animals for each group). (e) Representative traces of action potential firing from WT+SCR-shRNA, WT+5-HT_{2A}R-shRNA, WT+5-HT_{1A}R-shRNA, APP/PS1+SCR-shRNA, APP/PS1+5-HT_{2A}R-shRNA and APP/PS1+5-HT_{1A}R-shRNA mice and statistical data for action potential firing recorded from six groups (n = 10-15 cells from 3-4 mice for each group). (f) Representative traces of eEPSCs from WT+SCR-shRNA, WT+5-HT_{2A}R-shRNA, WT +5-HT_{1A}R-shRNA, APP/PS1 +SCR-shRNA, APP/PS1+5-HT_{2A}R-shRNA and APP/PS1 +5-HT_{1A}R-shRNA mice and statistical data for eEPSCs recorded from six groups (n = 15-17 cells from 4 mice for each group). (g) Representative traces of sEPSCs from WT+SCR-shRNA, WT+5-HT_{2A}R-shRNA, WT+5-HT_{1A}R-shRNA, APP/PS1+SCR-shRNA, APP/PS1+5-HT_{2A}R-shRNA and APP/PS1 +5-HT_{1A}R-shRNA mice and statistical data for sEPSCs recorded from six groups (n = 14-17 cells from 4 mice for each group). (h) Representative traces of sIPSCs from WT +SCR-shRNA, WT+5-HT_{2A}R-shRNA, WT+5-HT_{1A}R-shRNA, APP/PS1+SCR-shRNA, APP/PS1+5-HT_{2A}R-shRNA and APP/PS1 +5-HT_{1A}R-shRNA mice and statistical data for sIPSCs recorded from six groups (n = 12-14 cell from 4

mice for each group). Significance was assessed by two-way repeated measures ANOVA with major effect in (e) and (f), with post hoc comparisons (Turkey test) between groups in (c), (d), (g) and (h). All data are presented as mean ± SEM. *$p < 0.05$; **$p < 0.01$.
